# Visualization of topological shear polaritons in gypsum thin films

**DOI:** 10.1126/sciadv.adw3452

**Published:** 2025-07-18

**Authors:** Pablo Díaz-Núñez, Christian Lanza, Ziwei Wang, Vasyl G. Kravets, Jiahua Duan, José Álvarez-Cuervo, Aitana Tarazaga Martín-Luengo, Alexander N. Grigorenko, Qian Yang, Alexander Paarmann, Joshua Caldwell, Pablo Alonso-González, Artem Mishchenko

**Affiliations:** ^1^ Department of Physics and Astronomy, University of Manchester, Manchester, UK.; ^2^ National Graphene Institute, University of Manchester, Manchester, UK.; ^3^ Department of Physics, University of Oviedo, Oviedo, Spain.; ^4^ Center of Research on Nanomaterials and Nanotechnology CINN (CSIC–Universidad de Oviedo), El Entrego, Spain.; ^5^ Center for Quantum Physics, Key Laboratory of Advanced Optoelectronic Quantum Architecture and Measurement (MOE), School of Physics, Beijing Institute of Technology, Beijing, China.; ^6^ Beijing Key Laboratory of Nanophotonics and Ultrafine Optoelectronic System, Beijing Institute of Technology, Beijing, China.; ^7^ Fritz Haber Institute of the Max Planck Society, Berlin, Germany.; ^8^ Department of Mechanical engineering, Vanderbilt University, Nashville, TN, USA.

## Abstract

Low-symmetry crystals have emerged as a platform for exploring unique light-matter interactions in the form of hyperbolic shear polaritons. These excitations exhibit unique properties such as frequency-dispersive optical axes and asymmetric light propagation and energy dissipation. However, only non-vdW materials have been demonstrated to support hyperbolic shear polaritons, limiting their exotic properties and potential applications. Here, we introduce shear phenomena in low symmetry crystals by demonstrating elliptical and canalized shear phonon polaritons in gypsum, an exfoliable monoclinic sulphate mineral. Our results unveil a topological transition from hyperbolic shear to elliptical shear polaritons, passing through a canalization regime with strong field confinement. We observe a notable slowdown of group velocity, reaching values as low as 0.0005*c*. These findings expand the application scope of low-symmetry crystals with the benefits that an exfoliable material provides, such as stronger field confinement, tunability, and versatility for its incorporation in complex photonic devices.

## Teaser

Gypsum thin films support shear phonon polaritons in hyperbolic, canalized, and elliptical regime.

## INTRODUCTION

Phonon polaritons (PhPs; light-matter hybrid quasiparticles arising from the coupling of infrared photons with lattice vibrations in polar crystals) in thin layers of van der Waals (vdW) materials have attracted enormous attention in recent years. The highly anisotropic crystal nature of these materials has enabled the visualization of PhPs exhibiting exotic optical phenomena at the nanoscale. For instance, materials such as hexagonal boron nitride (h-BN) (*1*–*6*), alpha-molybdenum trioxide (α-MoO_3_) (*7*–*10*), or alpha-vanadium pentoxide (α-V_2_O_5_) (*11*–*13*) display hyperbolic PhPs, characterized by high momentum waves, subdiffractional light confinement, and anisotropic or highly directional light propagation. This hyperbolicity occurs in the so-called Reststrahlen bands (RBs) (*14*), where the real part of the permittivity tensor components of the material, $R\left\{\;\varepsilon_{\mathrm{ii}}\;\right\}$ , along different optical axes has opposite signs. For example, in-plane hyperbolicity in α-MoO_3_ arises when the in-plane permittivity components $\varepsilon_{\mathrm{xx}}$ , $\varepsilon_{\mathrm{yy}}$ satisfy $R\left\{\;\varepsilon_{\mathrm{xx}}\;\right\}\cdot R\left\{\;\varepsilon_{\mathrm{yy}}\;\right\}<0$ (*7*, *9*). The hyperbolic regimes in these materials lead to a variety of exotic light propagation phenomena, such as negative reflection (*15*) and refraction (*16*, *17*), light canalization (*18*, *19*), subdiffractional imaging and focusing (*2*, *20*), and strong coupling (*21*–*25*). Recently, studies on polaritons in low-symmetry crystals have led to the discovery of an entirely new type of hyperbolic PhPs, referred to as hyperbolic shear phonon polaritons (ShPhPs), which have been characterized experimentally in beta-gallium oxide (β-Ga_2_O_3_) (*26*, *27*) and cadmium tungstanate (CdWO_4_) (*28*) and theoretically investigated in artificial metasurfaces (*29*). Unlike high-symmetry crystals with orthogonal axes, such as α-MoO_3_, these low-symmetry crystals have monoclinic structure with a non-orthogonal angle between the crystal axes in the monoclinic plane, allowing the existence of non-orthogonal resonances. As a result, the permittivity tensor of these crystals cannot be diagonalized and contains nonzero off-diagonal components (*30*). This gives rise to the key features of shear polaritons that include asymmetric propagation and asymmetric energy dissipation and rotation of the optical axis as a function of the excitation frequency and, hence, dispersion of the propagation direction of the polariton. These properties have opened up new avenues for exploring novel optical phenomena and potential applications in nano-optics (*31*, *32*), and they have even been extended to elastodynamic metasurfaces (*33*). However, and in contrast to orthogonal crystals, only non-vdW materials have been demonstrated to support hyperbolic shear polaritons, limiting their exotic properties and potential applications. Therefore, fundamental questions remain open, such as whether ShPhPs can exist in thin layers or if nonhyperbolic propagation is possible.

In this work, we report the first observation of elliptical shear and canalized ShPhPs in thin layers of gypsum (CaSO_4_·2H_2_O). By combining real-space nano-imaging and infrared nanospectroscopy (nano-FTIR) using scattering-type scanning near-field optical microscopy (s-SNOM), we directly visualize the transition from hyperbolic shear to elliptical shear polariton propagation, with shear canalization emerging between these two regimes. We observe these phenomena in an exfoliable vdW material, which allows strong polaritonic field confinement in thin layers. These findings open opportunities for integrating low-symmetry crystals into complex heterostructures, enabling the development of advanced nanophotonic devices using shear light-matter excitations. In this sense, despite having been found in an insulator, ShPhPs in gypsum can be coupled to, for example, plasmons in graphene, which would establish an electrically tuneable platform to study unprecedented light-matter interactions and wave propagation. This platform would depend on different complementary parameters, such as Fermi energy, twist-angle between layers, or flake thickness. Moreover, these findings are relevant to the development of non-Hermitian nanophotonics applications.

## RESULTS

### Crystal structure and infrared response of Gypsum

**Fig. 1.**
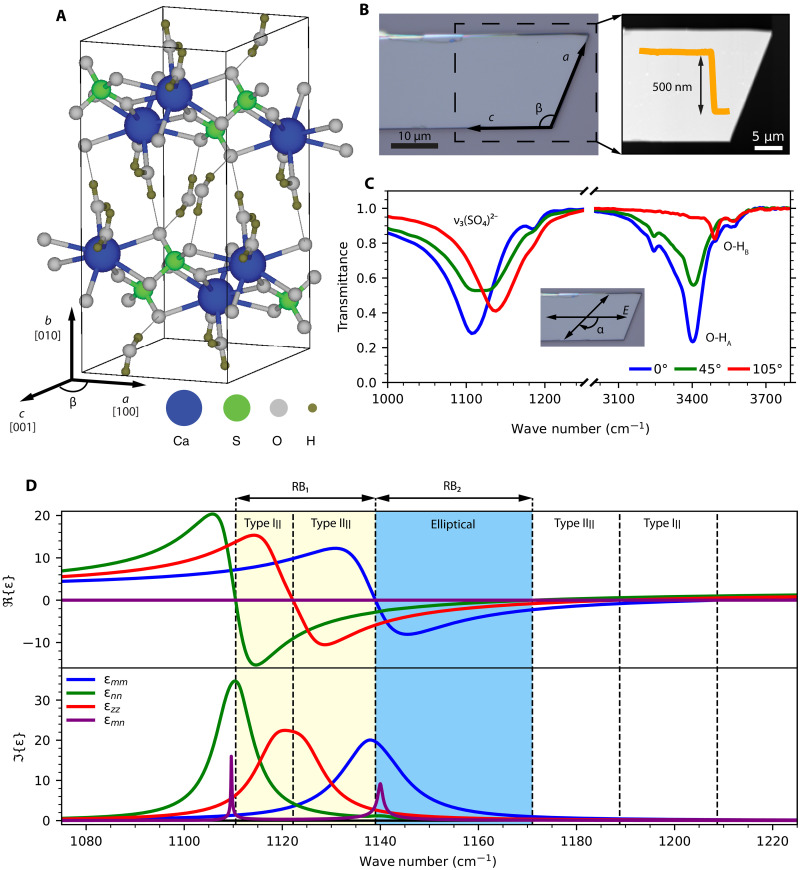
Crystallographic and optical properties of gypsum. (**A**) Crystal structure of gypsum. (**B**) Optical image and AFM topography of a 500-nm-thick gypsum flake. (**C**) Infrared transmittance spectrum of the flake in (B) at different polarization angles with respect to the *c* axis. The inset shows the orientation of the polarization with respect to the gypsum crystal axes: For α = 0, the electric field is parallel to the long axis of the flake and is rotating in clockwise direction. (**D**) Dielectric permittivity of gypsum in the frequency-dispersive coordinate system [mnz], exhibiting several RBs.

Gypsum (calcium sulphate dihydrate, CaSO_4_·2H_2_O) is one of the most abundant sulfate minerals in nature, with numerous industrial applications ranging from construction (*34*, *35*) to agriculture (*36*). The schematic in Fig. 1A shows its monoclinic crystal structure (space group I2/a ) with different lattice constants a=5.67Å , b=15.15Å , c=6.28Å , and monoclinic angle β≈114° between *a* and *c* axes (*37*–*40*). The crystal structure of gypsum consists of stacked bilayers along the *b* axis, with Ca^2+^ cations bound to SO_4_^2−^ anionic groups exhibiting a twofold axis symmetry. Between these layers, there is a bilayer of water molecules coordinated to a Ca^2+^ cation, forming two nonequivalent hydrogen bonds with the sulfate oxygen groups, and asymmetric in the crystal structure. This weak hydrogen bonding layer facilitates the cleavage of gypsum on the (010) plane (*41*), allowing the exfoliation of flakes along the monoclinic plane (Fig. 1B).

The highly anisotropic crystal structure of gypsum is reflected in its optical properties, particularly in the mid-infrared spectral range, where the fundamental vibrations correspond to either the sulfate groups or the water molecules (Fig. 1C and fig. S1) (*39*, *42*–*47*). This work focuses on the asymmetric stretching ν_3_ of SO_4_^2−^ between 1100 and 1250 cm^−1^, which exhibits very low infrared transmission (Fig. 1C), indicating the presence of a RB. In addition, we see the optical anisotropy of the monoclinic plane exhibiting non-orthogonal oscillators. Particularly, when the light is polarized parallel to the horizontal long edge of the flake in Fig. 1C ( α=0 ), we can identify one phonon centred at 1110 cm^−1^. As we rotate the direction of polarization, we observe a second phonon centered at 1138 cm^−1^, which is maximized when light polarization is closely parallel to the short edge of the flake. The angle between the edges is ~114°, matching that of the monoclinic angle β for gypsum. Consequently, the long and short edges of the flake in Fig. 1B can be identified as *c* and *a* axes of the monoclinic plane, respectively. The description of the dielectric permittivity tensor in the monoclinic plane is complex (*30*, *48*, *49*), but the infrared properties of gypsum have been studied extensively in the past (*38*, *39*, *42*, *44*–*47*) and Aronson *et al*. (*38*) reported its complete dielectric permittivity tensor. To unambiguously characterize the polaritonic regimes in a monoclinic crystal, the off-diagonal elements in the permittivity tensor are required to be zero at all frequencies for a coordinate system. However, there is no coordinate system in which $R\left\{\;\varepsilon_{\mathrm{xy}}\;\right\}=0$ at all frequencies. To overcome this limitation, we switch to a frequency-dispersive coordinate system [mnz] by rotating the monoclinic plane by the frequency-dependent angle γ(ω) (*26*, *29*)

$$\begin{array}{c} \gamma \left(\omega \right)=\frac{1}{2}{\text{tan}}^{-1}\left(\frac{2R\left\{\;\varepsilon_{\mathrm{xy}}\left(\omega \right)\;\right\}}{R\left\{\;\varepsilon_{\mathrm{xx}}\left(\omega \right)\;\right\}-R\left\{\;\varepsilon_{\mathrm{yy}}\left(\omega \right)\;\right\}}\right) \end{array}$$ (1)

The rotated permittivity tensor is included in Fig. 1D, exhibiting $R\left\{\;\varepsilon_{\mathrm{mn}}\;\right\}=0$ and $I\left\{\;\varepsilon_{\mathrm{mn}}\;\right\}\neq 0$ and, according to the transmission data, two transverse optical (TO) phonons at ~1110 and ~1138 cm^−1^ for $\varepsilon_{\mathrm{nn}}$ and $\varepsilon_{\mathrm{mm}}$ , respectively. Normal to the monoclinic plane, there is a TO phonon at ~1124 cm^−1^ for $\varepsilon_{\mathrm{zz}}$ . Therefore, we can identify a remarkable variety of narrow-frequency polaritonic regimes based on the signs of $\varepsilon_{\mathrm{mm}}$ , $\varepsilon_{\mathrm{nn}}$ , and $\varepsilon_{\mathrm{zz}}$ as follows: hyperbolic in-plane type I (1110 to 1122 cm^−1^), hyperbolic in-plane type II (1122 to 1138 cm^−1^), elliptical (1138 to 1172 cm^−1^), hyperbolic in-plane type II (1172 to 1189 cm^−1^), and hyperbolic in-plane type I (1189 to 1209 cm^−1^). This description is in excellent agreement with our transmittance measurements and with Raman spectra extracted from the same flake (fig. S1B).

### Probing phonon polaritons in gypsum thin films

**Fig. 2.**
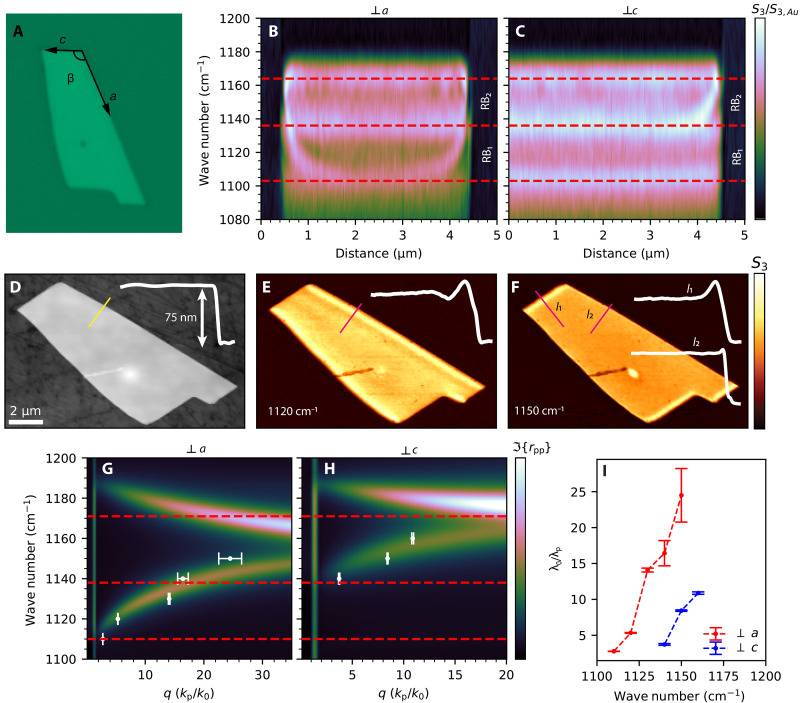
Near-field optical spectroscopy and imaging of PhPs in gypsum. (**A**) Optical image of a gypsum flake placed on a CaF_2_ substrate. (**B** and **C**) s-SNOM nano-FTIR line scans perpendicular to *a* and *c* gypsum crystal axes, respectively. (**D**) AFM topography of the gypsum flake with a thickness of 75 nm. (**E** and **F**) s-SNOM nano-imaging measurements at 1120 and 1150 cm^−1^, respectively. The insets in (D) to (F) represent the topography and near-field amplitude profiles along the lines in the corresponding panel. (**G** and **H**) PhP dispersion relation of a 75-nm-thick gypsum flake calculated with TMM perpendicular to *a* and *c* axes, respectively. The experimental values obtained from the fittings of monochromatic s-SNOM images are shown as white dots with their corresponding error bars. (**I**) PhPs confinement factors extracted perpendicular to *a* and *c* axes.

Motivated by these optical findings, we prove the potential excitation of polaritons in gypsum by performing s-SNOM nano-FTIR spectroscopy and real-space imaging on a 75-nm-thick gypsum flake exfoliated on a CaF_2_ substrate (Fig. 2A). In s-SNOM, the electric field confined at a metallic atomic force microscopy (AFM) tip provides enough momenta to launch and probe PhPs in polar crystals. In particular, the launched PhPs propagate until they reflect back at edges or discontinuities, forming a standing wave and generating interference fringes that can be detected by either nano-FTIR spectroscopy or monochromatic nano-imaging (*1*, *3*, *7*, *13*, *17*, *50*, *51*).

First, we acquired two broad-band nano-FTIR linescans perpendicular to the right and top edges of the flake, corresponding to the *a* and *c* axes of the gypsum crystal, respectively (Fig. 2, B and C). The resulting hyperspectral images show three distinct maxima as a function of distance from the edge at about 1105, 1135, and 1165 cm^−1^, as marked by the dashed lines in Fig. 2 (B and C), revealing two RB: RB_1_ and RB_2_. The first two maxima at ~1105 and ~1135 cm^−1^ can be identified as the two TO phonons in the monoclinic plane, in good agreement with the infrared transmittance and the dielectric permittivity shown in Fig. 1D. The third peak at 1165 cm^−1^ matches the longitudinal optical (LO) phonon along *nn* direction of the rotated frame, separating the elliptical dispersion from that with in-plane type II hyperbolic dispersion (Fig. 1D). We observe distinct signal maxima with strong spectral dispersion, i.e., resonances that vary with frequency as a function to the distance from the edge. As previously reported, this behavior unveils the propagation of PhPs (*1*, *7*, *13*), which we observe in both RB_1_ (between 1105 and 1135 cm^−1^) and RB_2_ (between 1135 and 1165 cm^−1^). In RB_1_, the signal maxima are only present in the scan perpendicular to the *a* axis, suggesting the excitation of in-plane hyperbolic PhPs (*7*). In contrast, RB_2_ exhibits signal maxima along the two in-plane axes, although more pronounced in the scan normal to the *c* axis, which suggests the excitation of in-plane elliptical PhPs (*7*). For both RBs, the PhP frequency increases closer to the edge, indicating a positive phase velocity (*1*, *7*, *13*).

To further analyze these observations, we performed s-SNOM nano-imaging using monochromatic illumination in the spectral range of 1100 to 1190 cm^−1^. The resulting images show the formation of signal maxima (fringes) parallel to the edges of the flakes (Fig. 2, E and F, and fig. S4), confirming the excitation of propagating PhPs in gypsum (*1*, *3*, *7*, *13*, *17*, *50*, *51*). In RB_1_ (Fig. 2E), the PhP fringes are only observed propagating perpendicular to the *a* axis, while no detectable fringes are seen perpendicular to the *c* axis. This observation demonstrates the excitation of PhPs with in-plane hyperbolic propagation. In RB_2_ (Fig. 2F), the fringes are visible propagating perpendicular to both edges, although with a clear difference in intensity and width (related to the PhPs wavelength), which confirms the excitation of PhPs with elliptical propagation. From the near-field raster scans, we extracted amplitude and phase, $S_{n}$ and $\varphi_{n}$ , line profiles perpendicular to *a* and *c* axes, and constructed the complex-value near-field signal, $\sigma_{n}=S_{n}e^{i\varphi_{n}}$ . We fitted $\sigma_{n}$ according to

$$E\left(x\right)=A\frac{e^{i2k_{p}x}}{\sqrt{2x}}+C$$ (2)

which describes the electric field of a radially propagating damped wave (*52*, *53*), with *A*, *k*_*p*,_ and *C* as fitting complex parameters (fig. S5). The extracted values of *k*_*p*_, representing the complex polariton wave vector, are plotted in Fig. 2(G and H) for PhPs propagating perpendicular to the *a* and *c* axes in gypsum, respectively. The dispersion relations of surface polaritons in a 75-nm-thick gypsum flake calculated by the transfer-matrix method (TMM) (*54*) are also plotted showing a good agreement with the experiment. The polariton wavelength can be calculated as $\lambda_{p}=2\pi /R\left(k_{p}\right)$ and the polariton propagation length as $L=1\;/\;I\left\{\;k_{p}\;\right\}$ . Thanks to the thin layer nature of the gypsum flakes, polariton confinements, $\lambda_{0}/\lambda_{p}$ , as large as 25 and 10 are obtained perpendicular to the *a* and *c* axes, respectively (Fig. 2I). In addition, we calculated the group velocity, $v_{G}=\partial \omega \;/\;\partial k$ , by fitting the experimental dispersion relation perpendicular to each crystal axis with a power law function of the form $y=ax^{b}$ and then performing its numerical derivative and the polariton lifetime as $\tau =L\;/\;v_{G}$ . We obtained values from 0.005*c* to 0.0005*c* with lifetimes between 2 and 0.6 ps for polaritons propagating perpendicular to the *a* axis in the frequency range between 1110 to 1150 cm^−1^. In the case of polaritons propagating perpendicular to the *c* axis, we obtained group velocities between 0.004*c* to 0.001*c* with a lifetime of around 0.8 ps in the frequency range between 1140 and 1160 cm^−1^ (fig. S5). These exceptionally low group velocities suggest the potential of gypsum films to harness “slow light” phenomena, facilitating enhanced light-matter interactions with possible applications in optical signal processing, nanoscale photonic circuits, and infrared sensing technologies (*55*–*58*).

Note that in our measurements, we only observe one or two polaritonic fringes, similar to previous observations of ultraconfined polaritons in Ag_2_Te, Bi_2_Se_3_, or monolayer h-BN (*52*, *53*, *59*). To quantify the propagation properties of PhPs in gypsum, we calculate the polariton inverse damping ratio (γ^−1^) and the relative propagation length ( $L\;/\;\lambda_{p}=\gamma^{-1}\;/\;2\pi$ ). In the hyperbolic regime, where a pair of polaritonic fringes is observed, we extract values of γ^−1^ and $L\;/\;\lambda_{p}$ around 4.5 to 6.5 and 0.75 to 1, respectively. Similarly, in the elliptical regime, we extract values of γ^−1^ and $L\;/\;\lambda_{p}$ of around 2 to 3 and 0.5, respectively (fig. S6). To better understand these metrics, we can express $L\;/\;\lambda_{p}$ as a function of the polariton wave vector $k_{p}$ , group velocity $v_{G}$ , and decay time τ (*52*, *53*)

$$\frac{L}{\lambda_{p}}=\frac{1}{2\pi }R\left\{\;k_{p}\;\right\}v_{G}\tau =\frac{\omega }{2\pi c}R\left\{\;q\;\right\}v_{G}\tau$$ (3)

With this expression, we can clearly see that, despite exhibiting relatively long lifetimes of 0.5 to 2 ps, the extremely small values of $v_{G}$ for PhPs in gypsum have a direct impact on their relative propagation length (fig. S6), explaining the observation of very few fringes in our s-SNOM experiments.

### Topological transition of ShPhPs in gypsum thin films

**Fig. 3.**
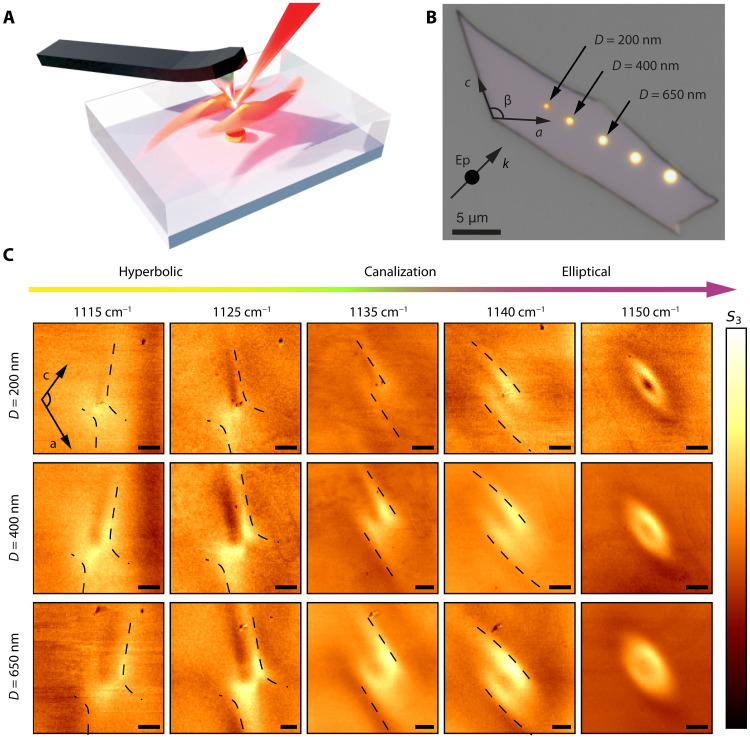
Near-field imaging of ShPhPs in gypsum: Transition from hyperbolic shear to elliptical shear passing through canalized shear propagation of PhPs. (**A**) Schematic representation of the s-SNOM near-field imaging of PhPs in a gypsum flake placed on top of an Au disk. (**B**) Optical image of the gypsum flake placed on the Au disks array. (**C**) s-SNOM images showing the near-field signal *S*_3_ at different incident frequencies (1115, 1125, 1135, 1140, and 1150 cm^−1^, from left to right panels) and for different disk diameters (200, 400, and 650 nm, from top to bottom panels). A clear transition from hyperbolic shear to elliptic shear propagation passing through canalized shear PhPs is observed. The thin dashed black lines are a guide to the eye in the hyperbolic and canalized regime.

To gain more insight on the propagation of PhPs in gypsum, we fabricated an array of Au disks on CaF_2_ (with diameters of 200, 400, 650, and 850 nm and 1 μm), transferred a 150-nm-thick gypsum flake on top of them, and performed s-SNOM near-field imaging (Fig. 3). This experiment allows us to directly visualize the directional propagation of PhPs because the circular contour of the disks allows them to be launched or reflected along all possible directions in the plane (in the case where the tip of the s-SNOM acts as launcher) (*20*, *27*, *32*, *60*). The main experimental results are summarized in Fig. 3C (full measurements are shown in fig. S7), where we plot images of the near-field amplitude, $S_{n}$ , demodulated at the *n =* 3 harmonic, as a function of illumination frequency and disk diameter. We observe asymmetric polaritonic patterns emanating from the disks that evolve from a hyperbolic to an elliptic contour with increasing frequency. This demonstrates a transition from hyperbolic to elliptical in-plane propagation of PhPs in gypsum. The asymmetry observed in the polaritonic patterns unveils a shear behavior, which, as highlighted in Fig. 1, has its origin in the nonzero off-axis components in the gypsum dielectric permittivity tensor (*26*, *28*). In the hyperbolic regime from 1110 to 1135 cm^−1^, we visualize shear asymmetric hyperbolas, which also display a notable rotation of their in-plane propagation as a function of frequency. This observation indicates a continuous dispersion of the optical axes, which has been previously reported for the bulk materials β-Ga_2_O_3_ and CdWO_4_ (*26*–*28*) and, more recently, in trigonal ReS_2_ and ReSe_2_ waveguide modes at near-infrared frequencies (*61*). When the excitation frequency matches the TO phonon at ~1138 cm^−1^, $R\left\{\;\varepsilon_{\mathrm{mm}}\;\right\}\sim \;0$ , the polaritonic signal consists of two parallel fringes (Fig. 3C; between 1135 and 1140 cm^−1^), unveiling a canalization effect in which the PhPs propagate along a single in-plane direction. This phenomenon occurs at the transition from hyperbolic to elliptical PhPs contours with increasing frequency (from left to right panels in Fig. 3C), thus revealing a “topological transition” from an opened to a closed isofrequency contour (IFC). Notably, in contrast to previous reports on canalization (*62*–*67*), canalized PhPs in gypsum show an asymmetric field intensity within their flattened wavefronts, thus unveiling their shear nature. Our near field images also reveal polaritonic contours consisting of elongated ellipsoids with asymmetric lobes, demonstrating the excitation of elliptical ShPhPs in gypsum (1150 cm^−1^; Fig. 3C), constituting the first observation of this type of highly asymmetric polaritons in a natural material. In addition, we include the Fourier transform of the real-space images in fig. S8.

**Fig. 4.**
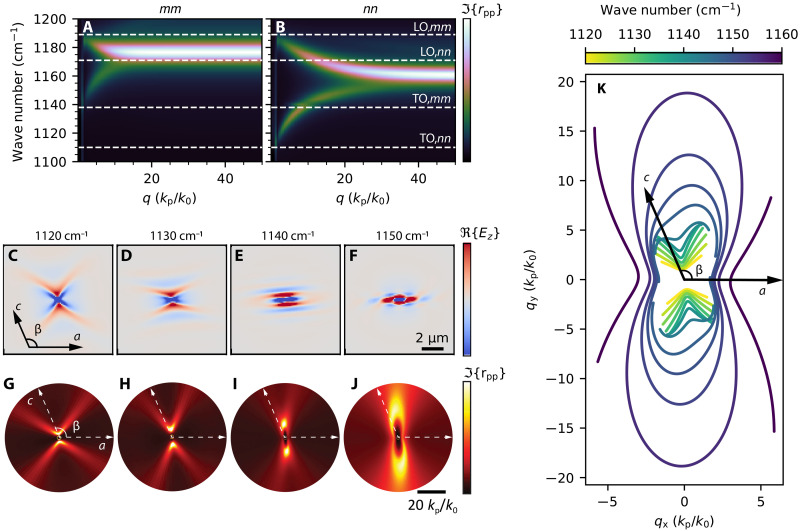
Theoretical dispersion and topological transition of ShPhPs in thin films of gypsum. (**A** and **B**) Dispersion of shear polaritons in 150-nm-thick gypsum along the *mm* (A) and *nn* (B) directions. (**C** to **F**) Real part of the out-of-plane electric field ( $R\left\{E_{z}\right\}$ ) of shear polaritons obtained with numerical simulations at 1120, 1130, 1140, and 1150 cm^−1^. (**G** to **J**) IFCs obtained with TMM at 1120, 1130, 1140, and 1150 cm^−1^, respectively. (**K**) Analytical IFCs at *q_z_* = 0 for PhPs in a 150-nm-thick gypsum layer.

To corroborate theoretically the polaritonic response observed in our thin films of gypsum, we conducted an analysis using TMM calculations (*54*). First, we verify the polaritonic behavior of gypsum by analyzing the polariton dispersion, ω(*q*), in the rotated coordinate system [mnz] along the *mm* (Fig. 4A) and *nn* (Fig. 4B) axes in the spectral range between 1100 and 1200 cm^−1^. We observe one polariton branch for each direction starting at different TO phonons. The TO phonon at ~1110 cm^−1^ along the *nn* direction produces the hyperbolic band, RB_1_, and, at ~1140 cm^−1^, another TO phonon is present along the *mm* direction, leading to the appearance of the elliptical band, RB_2_, matching our experimental observations. The elliptic RB exhibits a positive phase velocity along both crystal directions, in contrast to previous works (*7*, *13*), where elliptic polaritons showed a negative phase velocity. The dispersion is rather broad in the flat regime, which arises from a large damping value. Our theoretical analysis also confirms the subdiffractional nature of shear polaritons in gypsum (*q* > 1) along both axes, providing valuable insights into the unique polaritonic characteristics of thin gypsum layers. We also conducted full-wave numerical simulations of shear polaritons in a thin layer (*d* = 150 nm) of gypsum at four different frequencies: 1120, 1130, 1140, and 1150 cm^−1^, which are shown in Fig. 4 (C to F). In addition, the IFCs obtained with the TMM method (*54*) are shown in Fig. 4 (G to J). In agreement with the dispersion shown in Fig. 4 (A and B) and the experimental results shown in Fig. 3, we observe hyperbolic shear PhPs at 1120 and 1130 cm^−1^, which display an asymmetric hyperbolic contour. We also observe a canalized shear propagation at 1140 cm^−1^. This exotic canalization can be understood by examining the IFC, which displays a flat contour with an asymmetric intensity distribution, indicating that the flux of energy points along the same direction for all the allowed wave vectors, but with the asymmetric loss redistribution characteristic of shear polaritons (*27*–*29*, *33*). This peculiar propagation corresponds to the transition between $R\left\{\;\varepsilon_{\mathrm{mm}}\;\right\}>0$ and $R\left\{\;\varepsilon_{\mathrm{mm}}\;\right\}<0$ , which matches the behavior observed in the experimental result shown in Fig. 3C. Also, we demonstrate elliptical PhP propagation at 1150 cm^−1^. Last, to better visualize the topological transition between shear propagation regimes in gypsum, we plot in Fig. 4K the analytical IFCs in the (*q_x_*,*q_y_*) plane for *q_z_ =* 0 as a function of frequency. We observe the transition from hyperbolic shear PhPs (open IFCs in yellow) to canalized shear PhPs (dark green) and to elliptical shear PhPs (closed IFCs in blue), in good agreement with our numerical results and, more importantly, with our experimental images in Fig. 3.

The observed topological transition in gypsum phonon polaritons is qualitative equivalent to a Lifshitz transition for electrons (*68*). To quantify a topological transition in polaritonic systems, several topological invariants have been considered in the literature, for example, the number of anti-crossing points of the dispersion lines in twisted bilayers (*63*, *69*, *70*), which cannot be applied in our system. In gypsum polaritons, the transition is governed by the evolution of $R\left\{\;\varepsilon_{\mathrm{mm}}\;\right\}$ with frequency, equivalent to what has been reported in hyperbolic metasurfaces (*71*, *72*). Therefore, we have considered the Euler characteristic (or Euler number), which is a topological invariant that describes the topological shape or curvature. The Euler number does not change under continuous deformations such as stretching or bending but changes with tearing or gluing. It can be calculated as χ = *C – H*, where *C* represent the number of connected regions and *H* the number of holes. Therefore, for hyperbolic contours χ = 2, one for each branch, meanwhile for closed ellipsoidal contours, χ = 0. In addition. we have characterized the transition as a function of $\surd |\;-\;R\left\{\;\varepsilon_{\mathrm{mm}}\;\right\}\;/\;R\left\{\;\varepsilon_{\mathrm{nn}}\;\right\}\;|$ and its derivative, where we have included the absolute value to avoid complex numbers. The results are included in fig. S9. For frequencies smaller than 1139 cm^−1^, we are in the hyperbolic regime, therefore χ = 2, and the IFCs show the open hyperbolic branches. When $R\left\{\;\varepsilon_{\mathrm{mm}}\;\right\}=0$ at 1139 cm^−1^ (the TO phonon along mm), $\surd |\;-\;R\left\{\;\varepsilon_{\mathrm{mm}}\;\right\}\;/\;R\left\{\varepsilon_{\mathrm{nn}}\right\}\;|=0$ and its derivative presents a discontinuity. At this point, the transition begins, and the isofrequency curves start to bend backward. The Euler number does not change because the two branches are still open, but the shape rapidly evolves showing the parallel contours characteristic of canalization. With increasing frequency, the contours start to close and χ changes to 0 between 1148 and 1149 cm^−1^, showing the topological transition and closed contours.

## DISCUSSION

In this work, we have introduced gypsum as a material platform supporting unique polaritonic excitations such as elliptical shear, canalized shear, and hyperbolic shear PhPs. These shear optical phenomena are visualized in thin films of an exfoliable crystal, holding great potential against non-vdW materials in terms of increased field confinement, versatility, and the potential to leverage “slow light” phenomena due to low group velocities. These distinct polaritonic propagations arise from the very diverse and complex infrared permittivity tensor of gypsum within a narrow frequency range. In addition, the near-field experiments performed in thin layers of gypsum show the expected characteristics from shear polaritons, which include the asymmetric light propagation and loss redistribution, and dispersion of the optical axes.

When comparing the experimental results with the TMM results, we observe that the shear phenomena and the rotation of the hyperbola axis are larger in the experiments than in the calculations. The intensity of the asymmetric propagation and loss redistribution in shear polaritons have been demonstrated to be dependent on two parameters: the relative orientation angle between the oscillators and the losses (*28*, *29*, *33*). In gypsum, we are in a situation where, although the monoclinic angle is 114°, the angle between the oscillators is ~95° (*38*). This angle is very close to 90°, and therefore, it would induce a small shear effect (*29*). In addition, the axial dispersion, calculated by γ(ω) , varies about 3° between 1110 and 1135 cm^−1^ (fig. S2), and the rotation of the hyperbolic fringes is larger than 3° in the near-field imaging experiments at the same frequency range (Fig. 3 and fig. S6). However, the derivation of γ(ω) considers a lossless scenario (*29*), and it may be valid only when the losses are small (*38*, *73*). Also, the potential differences between the permittivity in (*38*) and the permittivity of our gypsum samples might have an impact between the theoretical and experimental results. These discrepancies do not affect our findings and should be studied elsewhere.

Although gypsum is unstable under certain conditions of temperature and humidity, its layered structure allows its encapsulation between h-BN protecting it from the environment even at cryogenic temperatures and high vacuum (*74*). Its monoclinic structure is of relevance to study low-symmetry nanophotonic phenomena from a fundamental point of view and for specific applications, especially in non-Hermitian photonics, or boundary states in the continuum (BIC). BIC are supported by non-orthogonal strongly couple modes (*75*, *76*), with potential applications in enhanced sensing. The non-orthogonality of optical phonons in gypsum makes it then ideal to study strongly confined BIC modes (*77*–*79*) and even has the potential to study polariton condensation phenomena (*80*). In addition, the remarkable slow group velocity of gypsum polaritons makes it an interesting platform for slow-light applications. One way to potentially harvest slow-light is through exceptional points (EPs) in non-Hermitian systems that obey parity-time (PT) symmetry (*81*–*85*). PT symmetry systems operate with a finely-tuned balance between gains and losses. The layered structure of gypsum gives the possibility of building metamaterials and heterostructures with alternating layers of gypsum and active materials to provide gain in hybrid photonic-phononic architectures (*86*). In addition, EPs in gypsum nanoresonators or gratings could have the potential to control wave transmission (*87*, *88*) or could be exploited for enhanced sensing (*89*–*91*).

In summary, our work paves the way for incorporating layered monoclinic crystals supporting ShPhPs into complex heterostructures and photonic devices. We envision that the coupling of shear polaritons with plasmons or other phonon polaritons is a promising route to unlock new non-Hermitian optical phenomena and a unique route to control light-matter interactions and wave propagation at the nanoscale.

## MATERIALS AND METHODS

### Sample preparation

Gypsum flakes were mechanically exfoliated onto Si substrates using Nitto tape. The selected flakes were picked up using a polydimethylsiloxane/polypropylene carbonate (PPC) stamp fabricated on a glass slide, transferred to a calcium fluoride (CaF_2_) substrate with prepatterned Au antennas, and cleaned with acetone to eliminate the PPC residue. The antennas were fabricated using standard electron beam lithography with two-layer polymethyl methacrylate resist (495,000/950,000 3% in anisole for bottom/top layer, respectively). The metals (3-nm Cr/27-nm Au) were deposited by electron-beam evaporation, followed by a lift-off in acetone, and cleaning with isopropanol.

### Fourier-transformed infrared spectroscopy

FTIR transmittance spectra in the range 800 to 4000 cm^−1^ were recorded with a Bruker Vertex 80 spectrometer equipped with a Bruker Hyperion 3000 FTIR microscope and a nitrogen-cooled mercury cadmium telluride detector for middle infrared region. Gypsum flakes exfoliated on a CaF_2_ substrate were focused through the standard 20x infrared (IR) (numerical aperture, NA = 0.4) Schwarzschild objective. A polarizer (A 675-P) was used to control the angle between the crystal axis and incident polarization direction. The spectra were acquired by rotating the polarizer manually with 15° step. All spectra were normalized to that of the CaF_2_ substrate, collected with 4 cm^−1^ resolution, and averaged more than 256 scans.

### Infrared nano-imaging and nano-FTIR spectroscopy

For the nano-imaging experiments, we used an AFM-based commercial scattering-type scanning near-field optical microscope (s-SNOM, Neaspec GmbH). A Pt/Ir-coated AFM tip (Arrow NCPt, NanoWorld, tip size of ~25 nm and force constant of 42 N/m) oscillating at its resonance frequency (ohm ≈ 285 kHz) with a tapping amplitude of ~ 60 nm is illuminated with monochromatic p-polarized light using a tunable quantum cascade laser with a parabolic mirror, while it raster scans the sample. The tip backscattered-light is collected by the same mirror and demodulated at higher harmonics of the tip vibration frequency *n*Ω (*n* ≥ 3) with a pseudo-heterodyne Michelson interferometer, yielding background-free amplitude and phase images of the near-field signal, together with the sample topography. The laser power was smaller than 1 mW, and the signal-to-noise ratio was about 150 for the third harmonic. The single wavelength images do not require normalization.

The nano-FTIR measurements were performed in the same s-SNOM with standard Pt/Ir-coated AFM tips (Arrow NCPt, NanoWorld). The tip is illuminated with a p-polarized mid-infrared broadband difference frequency generation laser (covering a frequency range of about 850 to 1600 cm^−1^ with an average power of less than 1 mW) using a parabolic mirror. Amplitude and phase spectra are obtained by using an asymmetric-Fourier transform interferometer on the back-scattered light reflected on the tip. The signal is demodulated to higher harmonics of the tapping amplitude, *n*Ω (*n* ≥ 3), to achieve background-free detection. We did line scans with a length of 5 μm and a spatial resolution of 25 nm. The nano-FTIR spectra were acquired averaging three interferograms of 700 μm (yielding a spectral resolution of about 7 cm^−1^) with 2048 pixels and an integration time of 10 ms per pixel. The recorded spectra were normalized to that of an Au patch evaporated in the same substrate (Au/CaF_2_).

### Raman spectroscopy

Polarized angle-resolved Raman spectra were acquired using a Witec alpha300 R confocal microscope using an excitation wavelength of 532 nm in parallel configuration of the incident and scattered light, i.e., with polarizer and analyzer parallel to each other and rotated simultaneously, using a 100× objective and a diffraction grating of 600 grooves/mm. For the measurements in Fig. 1, the angle-resolved spectra were recorded with a step of 5°. For each angle, 15 spectra were averaged with a laser power of about 2 mW and integration time of 2 s per spectra. For the Raman spectra on fig. S3, the angle-resolved spectra were recorded with a step of 10° using an integration time of 500 s and a laser power of 4 mW for the 75-nm-thick flake and 5 mW for the 150-nm-thick flake. All the spectra were fitted using a series of Lorentzian functions.

### Transfer-matrix numerical simulations

The Transfer-matrix numerical approach (*54*) was used to predict the polariton dispersion of a thin layer of gypsum (150 nm) embedded between air and CaF_2_ and the IFCs at several frequencies. We computed the imaginary part of the reflection coefficient ( $I\left\{\;r_{\mathrm{pp}}\;\right\}$ ), whose poles determine the maxima of the color plots, corresponding to the polariton dispersion. The permittivity of gypsum has been obtained from (*38*). The permittivity of air is assumed to be the vacuum permittivity, while the permittivity of CaF_2_ is assumed to be 1.6 along the spectral range of interest.

### Full-wave numerical simulations

The full-wave numerical simulations were performed using the software COMSOL Multiphysics, which is based on the finite boundary elements method. The system is made of two quasi–semi-infinite media fulfilling the role of superstrate (air) and substrate (CaF_2_) and a thin layer of the monoclinic crystal gypsum with *d* = 150 nm. A vertical point dipole placed 100 nm over the surface of the gypsum layer is used as the launcher to excite PhPs.

## Supplementary Materials

### This PDF file includes:

Supplementary Text Tables S1 and S2 Figs. S1 to S10 References
