# Supplementary material for: Visualization of topological shear polaritons in gypsum thin films
Source: Sci Adv. 2025 Jul 18;11(29):eadw3452. doi: 10.1126/sciadv.adw3452 (PMC12273749; doi:10.1126/sciadv.adw3452)
Supplement: Supplementary file 1 — Supplementary Text Tables S1 and S2 Figs. S1 to S10 References [file sciadv.adw3452_sm.pdf]

Science Advances
AAAS

Supplementary Materials for

Visualization of topological shear polaritons in gypsum thin films

Pablo Díaz-Núñez *et al.*

Corresponding author: Pablo Díaz-Núñez, pablo.diaznunez@manchester.ac.uk;
Pablo Alonso-González, pabloalonso@uniovi.es; Artem Mishchenko, artem.mishchenko@manchester.ac.uk

Sci. Adv. **11**, eadw3452 (2025)
DOI: 10.1126/sciadv.adw3452

This PDF file includes:

Supplementary Text
Tables S1 and S2
Figs. S1 to S10
References

Supplementary Text

Analytical approximations to the dispersion of shear polaritons in thin

The analytical expression for the dispersion of shear-like polaritons in thin films can be approximated as:

$$q_p = \frac{\rho}{k_0 d}\left[atan\left(\frac{\varepsilon_S \rho}{\varepsilon_{zz}}\right) + atan\left(\frac{\varepsilon_s \rho}{\varepsilon_{zz}}\right) + \pi l\right],$$

where $q_p = \frac{k_p}{k_0}$ stands for the normalized in-plane wavevector, k_0 and d are the free-space light wavevector and flake thickness, respectively; ε_S and ε_s are the superstrate and substrate permittivity, and

$$\rho = \sqrt{-\frac{\varepsilon_{zz}}{\varepsilon_{xx}cos^2\varphi + \varepsilon_{yy}sin^2\varphi + 2\varepsilon_{xy}sin\varphi cos\varphi}},$$

with φ the in-plane angle with regards to the *xx* axis. A detailed proof of the former result will be given elsewhere.

However, in the diagonal coordinate system [mnz], where the absolute value of the off-diagonal terms $|\Im\{\varepsilon_{mn}\}|$ are small compared to the absolute value of the diagonal terms $|\{\varepsilon_{mm}\}|$ and $|\Im\{\varepsilon_{nn}\}|$ and $|\{\varepsilon_{zz}\}|$, the dispersion of polaritons along the *mm* and *nn* directions can be approximated by the high-momentum approximation (*92*) with

$$\rho = \sqrt{-\frac{\varepsilon_{zz}}{\varepsilon_{mm/nn}}},$$

for the *mm* and *nn* directions

Infrared dielectric permittivity tensor of gypsum

Aronson *et al.* (*38*) derived the infrared dielectric permittivity tensor from infrared reflection spectroscopy. They applied a series of Lorentz oscillators to fit the reflectance spectra at three different angles to obtain the dielectric tensor of a monoclinic crystal with incident light normal to the monoclinic plane and parallel to b axis. The general form of the dielectric tensor in a monoclinic crystal with non-zero components in the monoclinic plane (plane xy) in a cardinal system, [xyz], is as follows:

$$\overline{\overline{\varepsilon(\omega)}} = \begin{bmatrix} \varepsilon_{xx}(\omega) & \varepsilon_{xy}(\omega) & 0 \\ \varepsilon_{yx}(\omega) & \varepsilon_{yy}(\omega) & 0 \\ 0 & 0 & \varepsilon_{zz}(\omega) \end{bmatrix},$$

where:

$$\varepsilon_{xx}(\omega) = \varepsilon_{xx}^{\infty} + \textstyle\sum_k \cos^2(\theta_k) \cdot L_k^{mp}(\omega),$$
$$\varepsilon_{xy}(\omega) = \varepsilon_{yx}(\omega) = \varepsilon_{xy}^{\infty} + \textstyle\sum_k \cos(\theta_k)\sin(\theta_k) \cdot L_k^{mp}(\omega),$$
$$\varepsilon_{yy}(\omega) = \varepsilon_{yy}^{\infty} + \textstyle\sum_k \sin^2(\theta_k) \cdot L_k^{mp}(\omega),$$
$$\varepsilon_{zz}(\omega) = \varepsilon_{zz}^{\infty} + \textstyle\sum_k L_k^{||b}(\omega).$$

Where ε^{∞} are the high frequency permittivity values, θ is the orientation of the oscillator with respect to the a axis, and L_k^{mp} and $L_k^{||b}$ represent the Lorentz oscillators in the monoclinic plane and parallel to b axis, respectively. Each Lorentzian oscillator, *k*, is defined as:

$$L_k = \frac{S_k}{1 + i\gamma_k\left(\omega/\omega_k\right) - \left(\omega/\omega_k\right)^2},$$

where ω_k is the resonant frequency (TO phonon), γ_k is the damping constant, and S_k is the oscillator strength. The Lorentz oscillators are summarized in Table S1 and Table S2.

Table S1. Lorentz oscillator parameters for gypsum in the monoclinic plane (*38*).

Oscillator (*k*)	ω_k (cm^{-1})	γ_k	S_k	θ_k
1	3406.3	0.01064	0.04313	85.04
2	1619.8	0.00736	0.02079	77.59
3	1138.2	0.01295	0.26264	4.93
4	1110.2	0.00797	0.28429	99.87
5	667.4	0.01027	0.12195	-7.90
6	598.3	0.02559	0.16739	87.30
7	465.2	0.15923	0.92815	-19.05
$\varepsilon_{xx}^{\infty} = 2.2819$ $\varepsilon_{yy}^{\infty} = 2.4545$ $\varepsilon_{xy}^{\infty} = 0.0086$				

Table S2. Lorentz oscillator parameters for gypsum normal to the monoclinic plane (***E*** ∥ *b*) (*38*).

Oscillator (*k*)	ω_k (cm^{-1})	γ_k	S_k
1	3525.8	0.01768	0.05719
2	1684.5	0.00955	0.00716
3	1124.3	0.00985	0.13638
4	1118.6	0.00945	0.13921
5	601.3	0.05583	0.24471
6	544.9	0.02050	0.00798
$\varepsilon_{zz}^{\infty} = 2.223$			

We reproduce the infrared permittivity of gypsum in fig. S2A in the range 1080-1220 cm^{-1}. In the monoclinic plane, the dielectric function features two oscillators at ~1110 and ~1138 cm^{-1} for ε_{yy} and ε_{xx}, respectively with a non-zero off-axis component (ε_{xy}). Normal to the monoclinic plane there are two overlapping oscillators at ~1118 and ~1124 cm^{-1} for ε_{zz}. In the monoclinic plane, the oscillators at 1138 and 1110 cm^{-1} have an angle of ~5° and ~100° with respect to *a* axis, respectively. The crystal axis assignment in Aronson´s description (*38*) of the gypsum crystal structure is equivalent to that used in this work. However, other studies feature an opposite axis notation assignment, which does not affect the overall physical properties. For instance, Takahashi *et al.* (*45*) assign the first oscillator at 1135 cm^{-1} parallel to the *c* axis and the second oscillator at 1110 cm^{-1} orthogonal to the *c* axis.

To capture the rotation of the optical axes in the monoclinic plane Passler *et al.* (*26*) diagonalized the real part of the permittivity tensor individually at different frequencies by rotating the monoclinic plane using the frequency-dependent angle:

$$\gamma(\omega) = \frac{1}{2}\tan^{-1}\left(\frac{2\Re\{\varepsilon_{xy}(\omega)\}}{\Re\{\varepsilon_{xx}(\omega)\} - \Re\{\varepsilon_{yy}(\omega)\}}\right)$$

The rotation is performed applying a rotation matrix, $R(\theta)$, defined as:

$$R(\theta) = \begin{bmatrix} \cos\theta & \sin\theta & 0 \\ -\sin\theta & \cos\theta & 0 \\ 0 & 0 & 1 \end{bmatrix}$$

The rotated system, [mnz], is calculated as $\bar{\bar{\varepsilon}}_{[mnz]} = R(-\theta)\bar{\bar{\varepsilon}}_{[xyz]}R(\theta)$ with $\theta = \gamma(\omega)$. In the rotated coordinate system, the real part of ε_{mn} is equal to zero, however, the imaginary part cannot be diagonalized at the same time therefore exhibiting shear phenomena. In the rotated frame we can unambiguously identify the type of polariton propagation supported by gypsum using the real part of the diagonalized permittivity as hyperbolic type I (one element negative), hyperbolic type II (two elements negative), or elliptical (all elements negative). Hyperbolic modes are also classified as in- or out-of-plane.

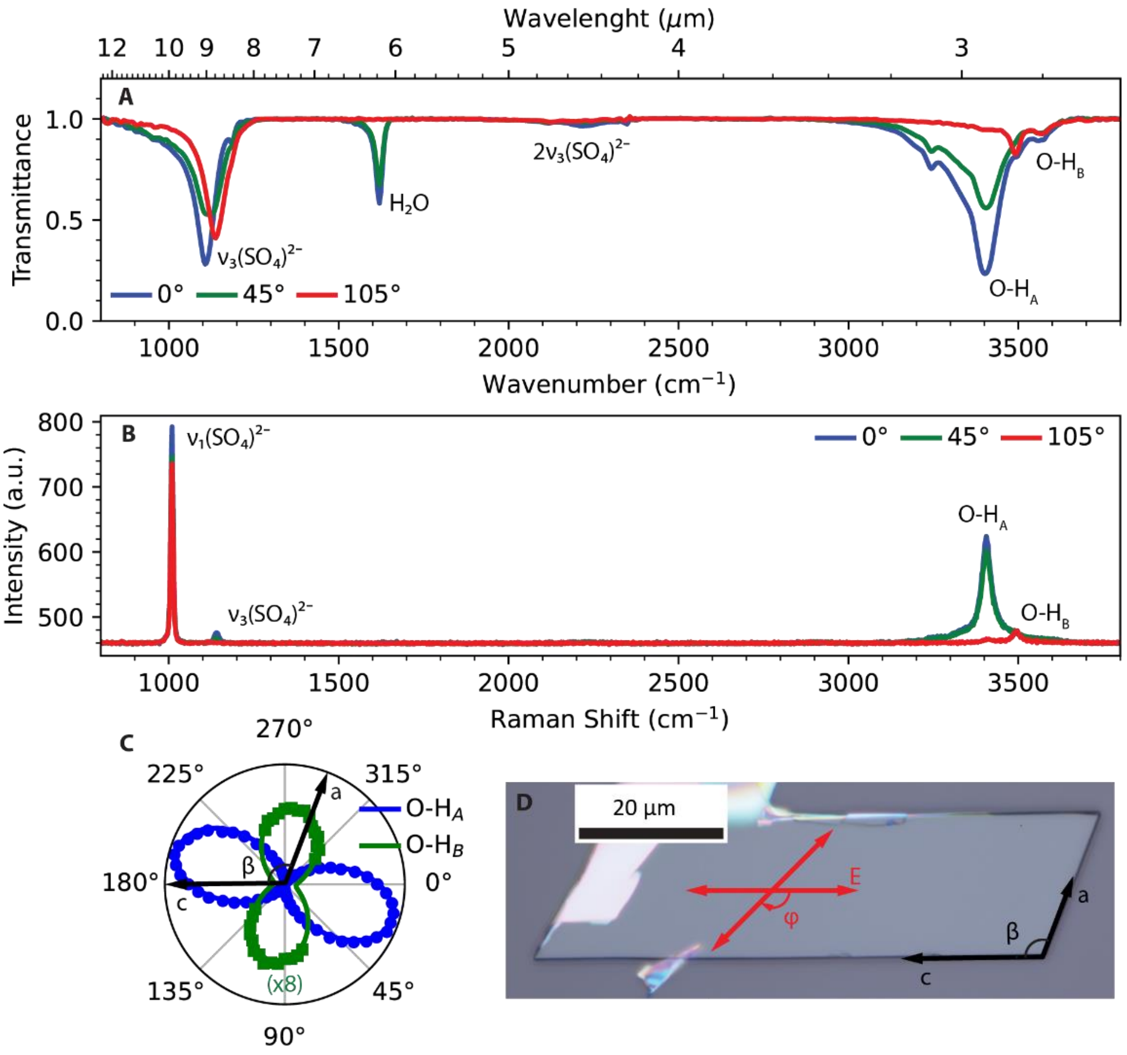

Fig. S1: Infrared response of gypsum in the monoclinic plane. (**A**) Polarized FTIR transmission and (**B**) Raman spectra in parallel configuration recorded at different angles with respect to the *c* axis of gypsum. (**C**) Raman polar plot of intensity of the stretching O-H bonds mode of water at ~ 3405 and 3495 $cm^{-1.}$ (**D**) Gypsum flake indicating the crystal axis in the monoclinic plane and the polarization. The observed fundamental normal vibrations correspond to the sulphate ionic group, $(SO_4)^{2-}$, and the water of crystallization molecules, H_2O. Regarding the sulphate vibrations, we first find the Raman-active symmetric stretching (ν_1) at ~1008 cm^{-1}, then the asymmetric stretching (ν_3) present in both Raman and infrared spectrum between ~1110 and 1140 cm^{-1}, and finally the first order overtones of the asymmetric stretching ($2\nu_3$) between 2100 and 2400 cm^{-1}. The water vibrations in gypsum vary from that of the free molecule because it is bonded to the crystal lattice, meaning that the two O-H bonds ($O\text{-}H_A$ and $O\text{-}H_B$) are not equivalent. In the FTIR spectra we find the infrared active bending mode of water that appears at ~1620 cm^{-1}. Note that there is another bending mode at ~1680 cm^{-1} that does not appear in the FTIR spectra in the monoclinic plane because it is an out-of-plane mode. The stretching of the $O\text{-}H_A$ and $O\text{-}H_B$ modes appear at ~3405 and 3520 cm^{-1}.

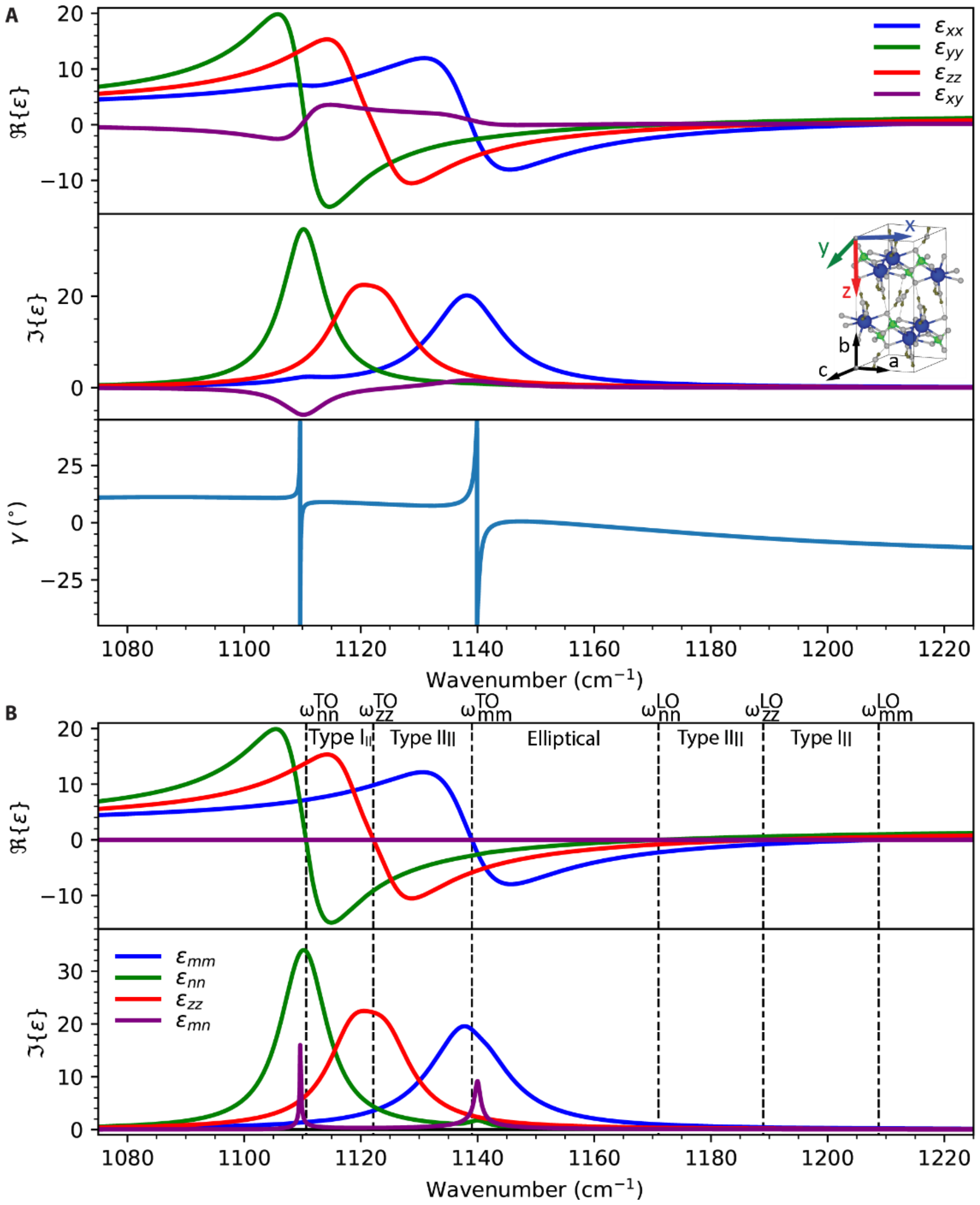

Fig. S2: Infrared permittivity tensor of gypsum in the range between 1075 and 1225 cm^{-1}. (**A**) Infrared dielectric permittivity tensor and rotation angle, γ, of gypsum reproduced from Aronson *et al*. (*38*). The inset represents the crystal structure with the coordinate system [xyz] in which the permittivity is expressed. (**B**) Dielectric permittivity tensor in the frequency-dispersive coordinate system [mnz] diagonalized by rotating the monoclinic plane by the frequency-dependent rotation angle γ. The real part of the off-axis components is zero, but the imaginary part is non-zero. Several RBs are identified as a function of the sign of the real part of ε_{mm}, ε_{nn} and ε_{zz}: type I and type II in-plane hyperbolic polaritons and elliptical polaritons.

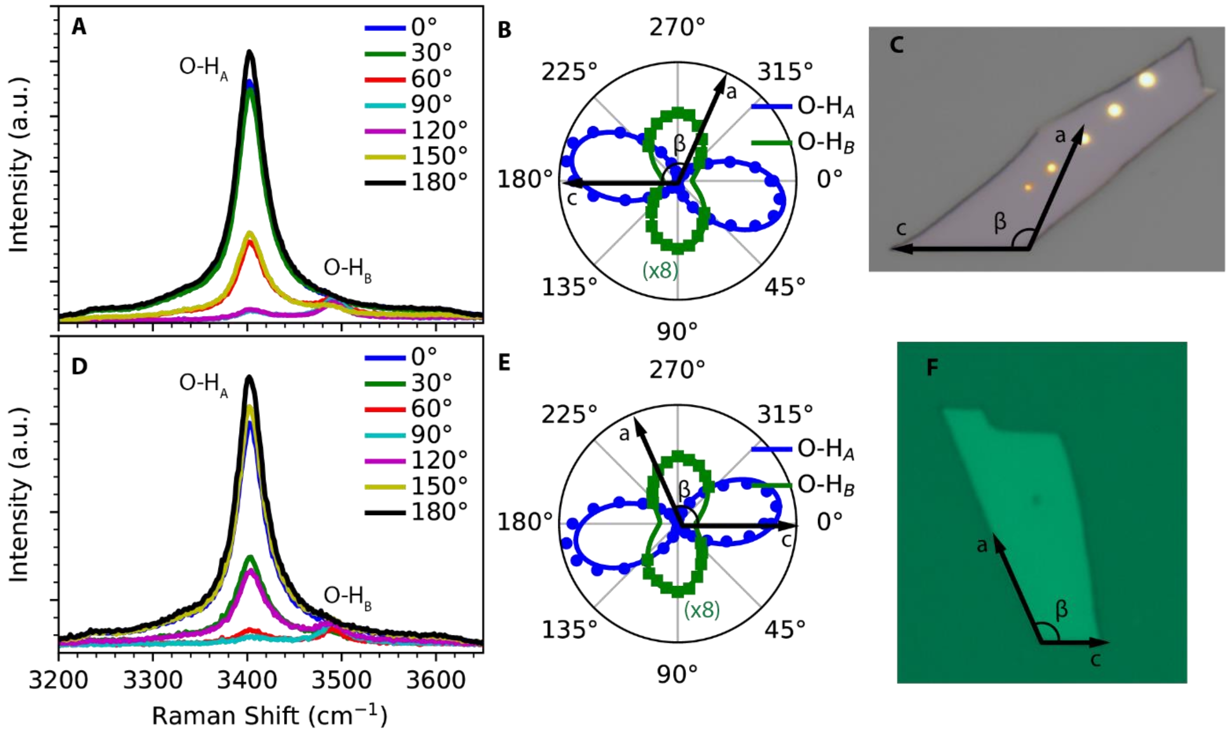

Fig. S3: Gypsum axis assignment based on polarized Raman spectroscopy of the stretching water modes. (**A,D**) Raman spectra of the stretching mode of water at different angles. (**B,E**) Experimental and fitting of the angular distribution of the O-H_A and O-H_B modes intensity. (**C, F**) Optical images of the gypsum samples indicating the crystal axis. We can identify the crystal axes of gypsum using the water molecules in the crystal structure applying angle-resolved polarized Raman spectroscopy with parallel configuration to probe the Raman-active OH^- stretching vibration. The splitting of the stretching mode of water in gypsum is attributed to two different hydrogen bonds that are present in the crystal structure (*47*) and well resolved at ~3405 cm^{-1} (O-H_A) and ~3480 cm^{-1} (O-H_B) (*39*, *41*, *42*, *44*).

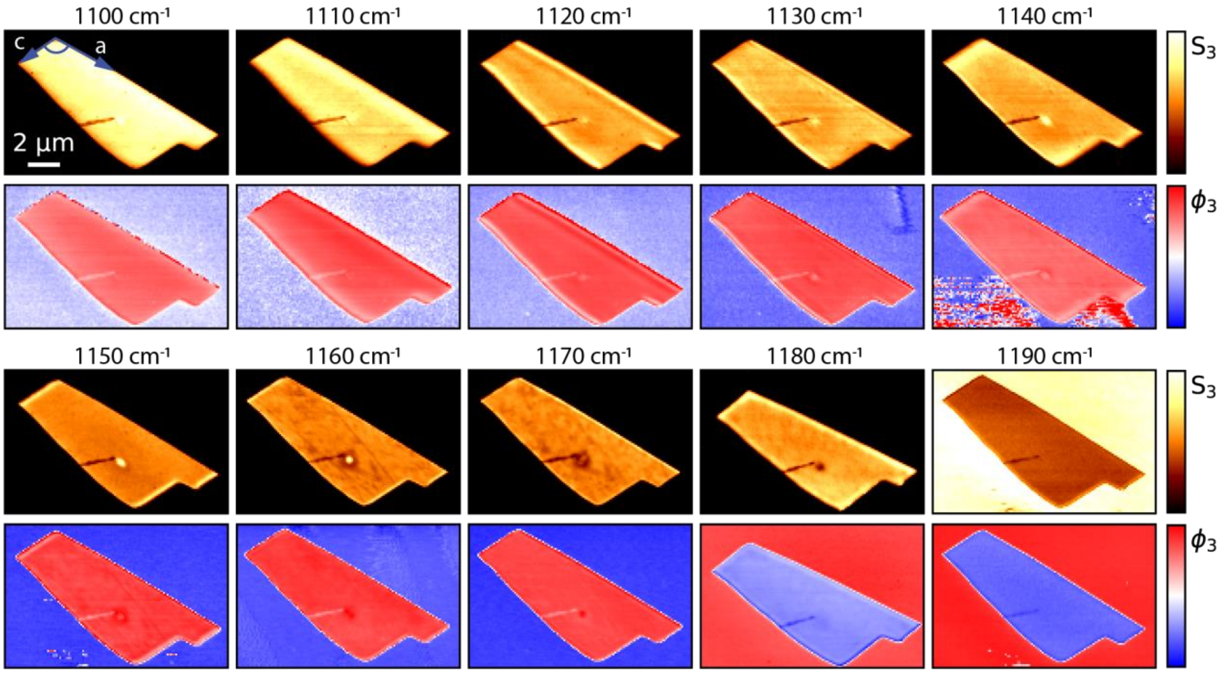

Fig. S4: Polariton interferometry of gypsum in the range 1100-1190 cm^{-1}. 3rd harmonic near-field amplitude (S_3) and phase (φ_3) raster scans of a gypsum flake with a thickness of 75 nm.

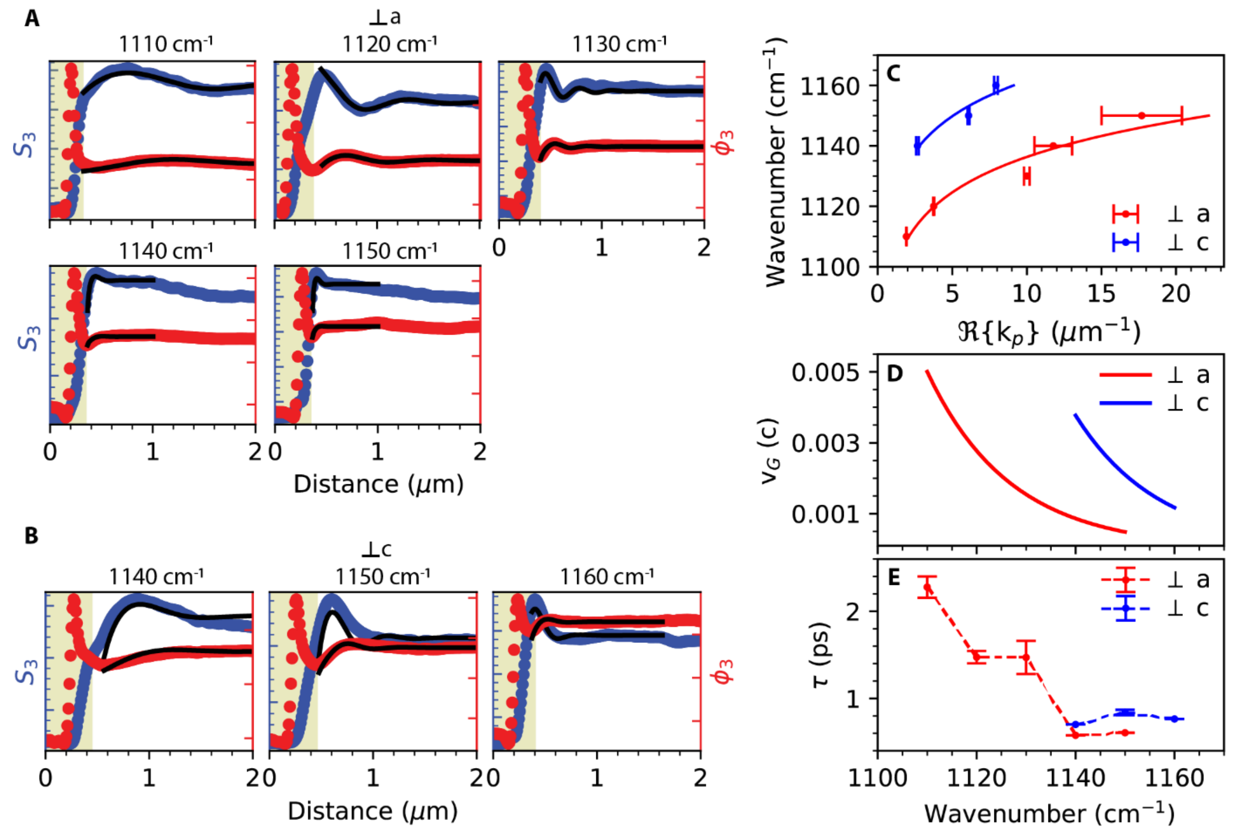

Fig. S5: Analysis of the near-field line profiles perpendicular to gypsum crystal axes. (**A,B**) 3rd harmonic near-field amplitude (S_3) and phase (φ_3) line profiles perpendicular to *a* and *c* axes, respectively, at selected frequencies extracted from the near-field images of fig. S4 and fitted according to Eq. 2. The dots represent the experimental measurement and the black lines the best fit. The pale-yellow rectangles in (A) and (B) indicate the substrate position and edge of the flake, which is not taken into account for the fitting. (**C**) Experimental dispersion relation (dots) extracted from the fits in (A) and (B) and fitted with a power law of the form $y = ax^b$ (lines) for polaritons propagating perpendicular to *a* and *c* axes. (**D**) Frequency-dependent group velocity of gypsum polaritons calculated as the numerical derivative of the fitted equation in (C). (**E**) Frequency-dependent polariton lifetime calculated as $\tau = L/v_G$.

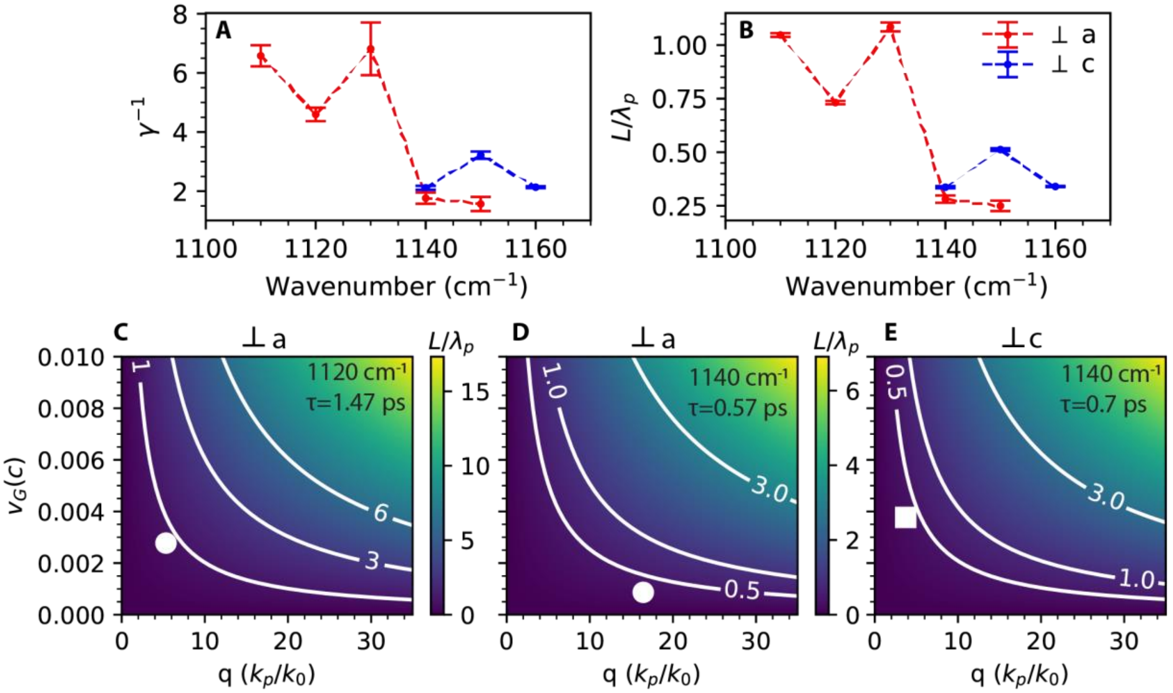

Fig. S6: Inverse damping rate and relative propagation length of phonon polaritons in gypsum. (A) Inverse damping constant and **(B)** relative propagation length for phonon polaritons in gypsum propagating perpendicular to *a* and *c* crystal axes. Relative propagation lengths for polaritons propagating **(C)** perpendicular to a axis at 1120 cm^{-1} and a decay time of 1.42 ps, **(D)** perpendicular to a axis at 1140 cm^{-1} and a decay time of 0.57 ps, and **(E)** perpendicular to c axis at 1140 cm^{-1} and a decay time of 0.7 ps. The single dots represent the experimental measurements of a 75 nm-thick gypsum flake.

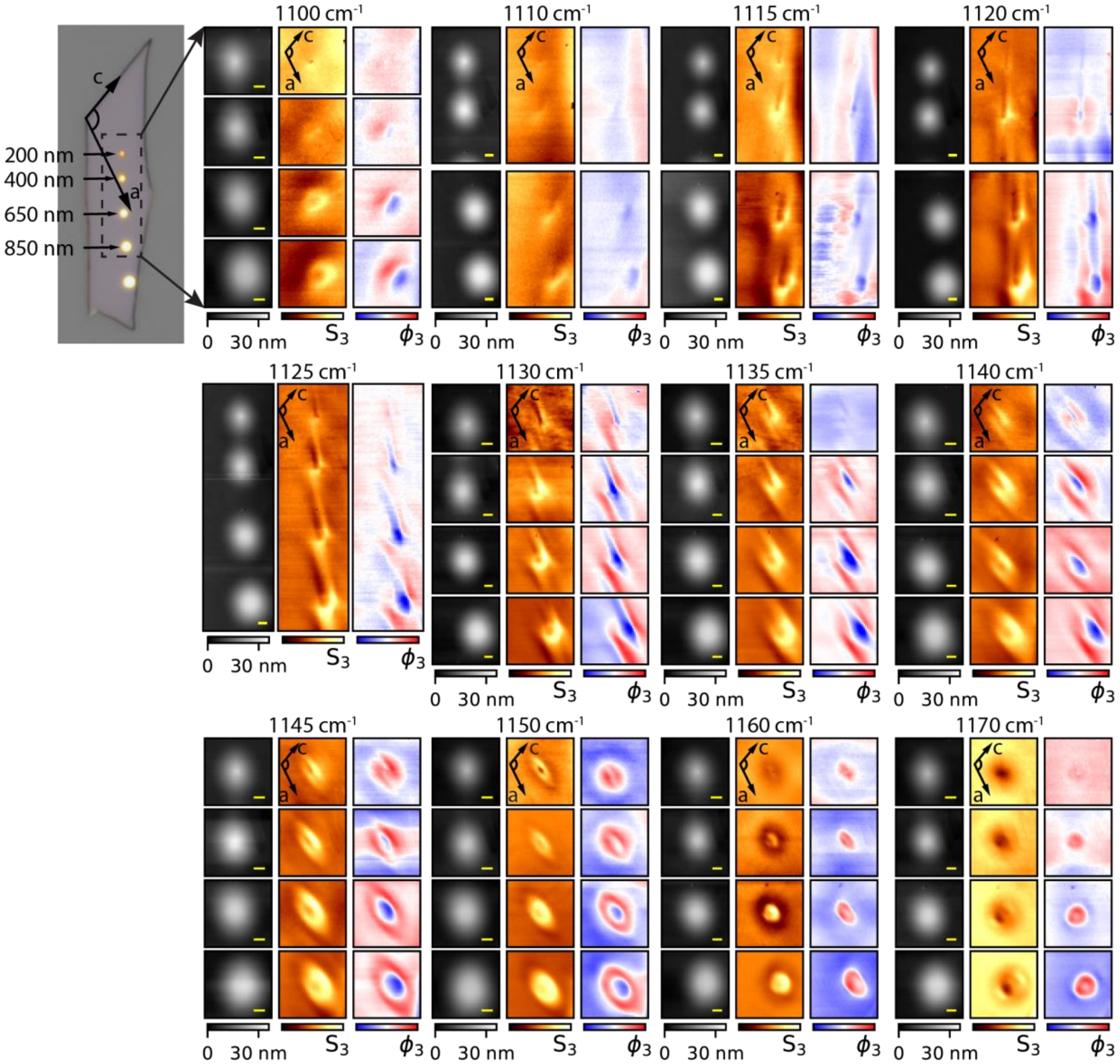

Fig. S7: Real-space observation of shear hyperbolic to shear elliptical transition on gypsum. Optical image (top left) and s-SNOM measurements on the first four Au disks of increasing diameter in the range 1100-1170 cm^{-1}. For each frequency we include the topography, near-field amplitude (S_3), and phase (φ_3). The shear hyperbolic fringes are clearly visualized starting from 1110 cm^{-1} showing the rotation of the fringes as the frequency is increased. The polaritons canalize at 1135-1140 cm^{-1}, and close showing shear elliptical polaritons between 1140 to 1150 cm^{-1}. The yellow scale bar in the AFM topography maps is 500 nm.

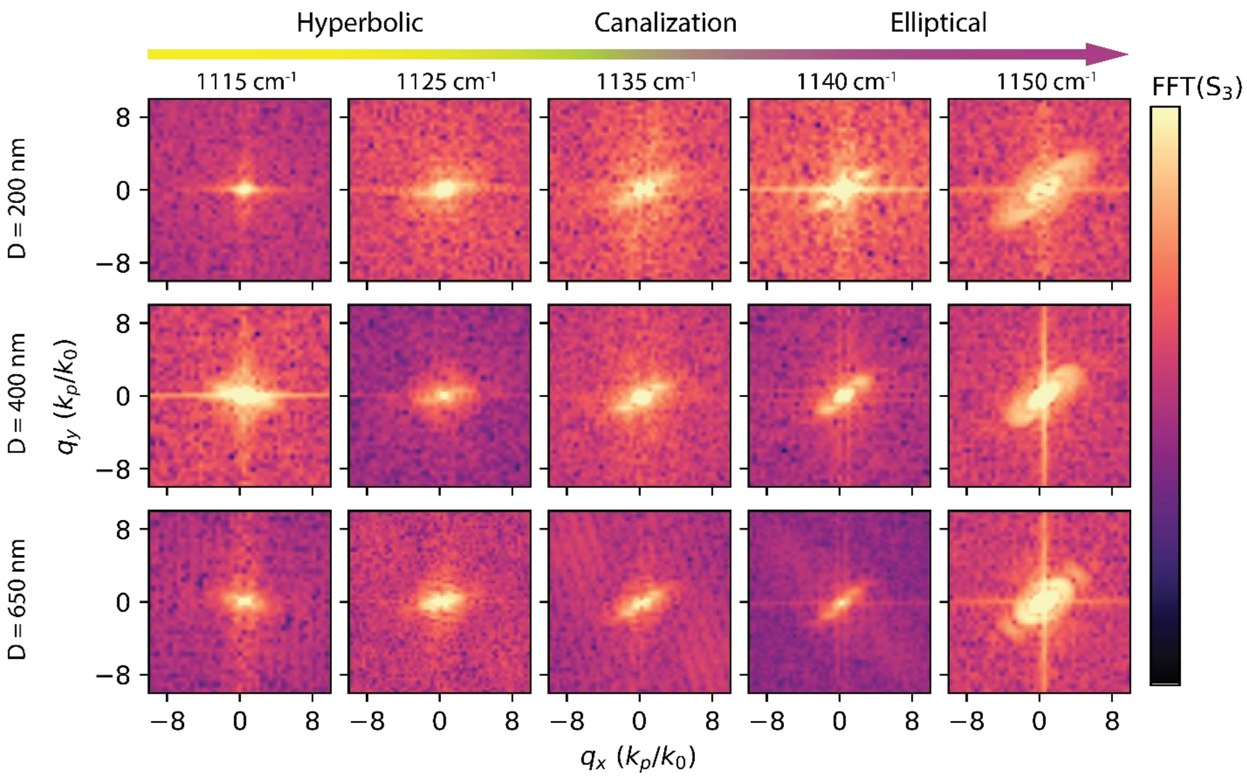

Fig. S8: Fourier transform of the real-space images. Fast Fourier transform (FFT) images of the real-space images of Fig. 3 at different incident frequencies (1115, 1125, 1135, 1140, and 1150 cm^{-1}, from left to right panels) and for different disk diameters (200, 400, and 650 nm, from top to bottom panels). The images are in very good agreement in the elliptical and canalized regimes (1135-1150 cm^{-1}). At 1135 cm^{-1} we can see the asymmetric parallel lobes characteristic of shear canalization. At 1140 cm^{-1} the two lobes start to bend backwards, and finally at 1150 cm^{-1} they closed, showing clear shear elliptical geometry. The uncertainty for the images in the hyperbolic regime (1115 and 1125 cm^{-1}) is attributed to the large noise after the transformation in small wavevectors.

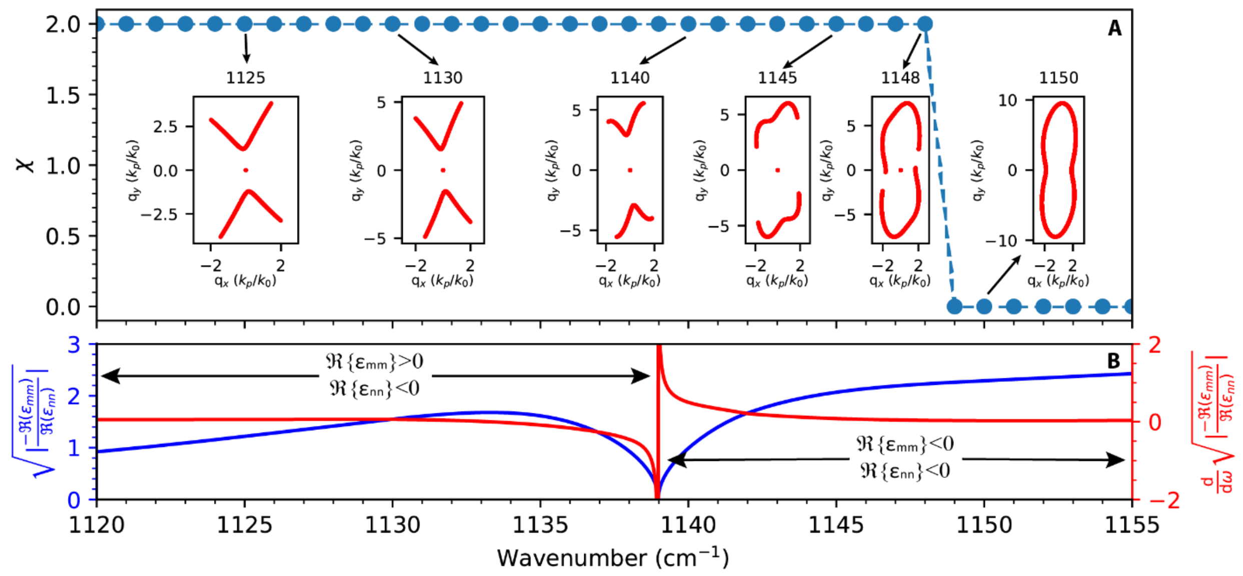

Fig. S9: Topological transition of shear phonon polaritons in gypsum: (A) Euler characteristic of the IFC as a function of the frequency. The insets represent the IFCs at selected frequencies. **(B)** Relationship $\sqrt{(|-\Re\{\varepsilon_{mm}\}/\Re\{\varepsilon_{nn}\}|)}$ (left axis) and its derivative (right axis), showing the transition point when $\Re\{\varepsilon_{mm}\} = 0$.

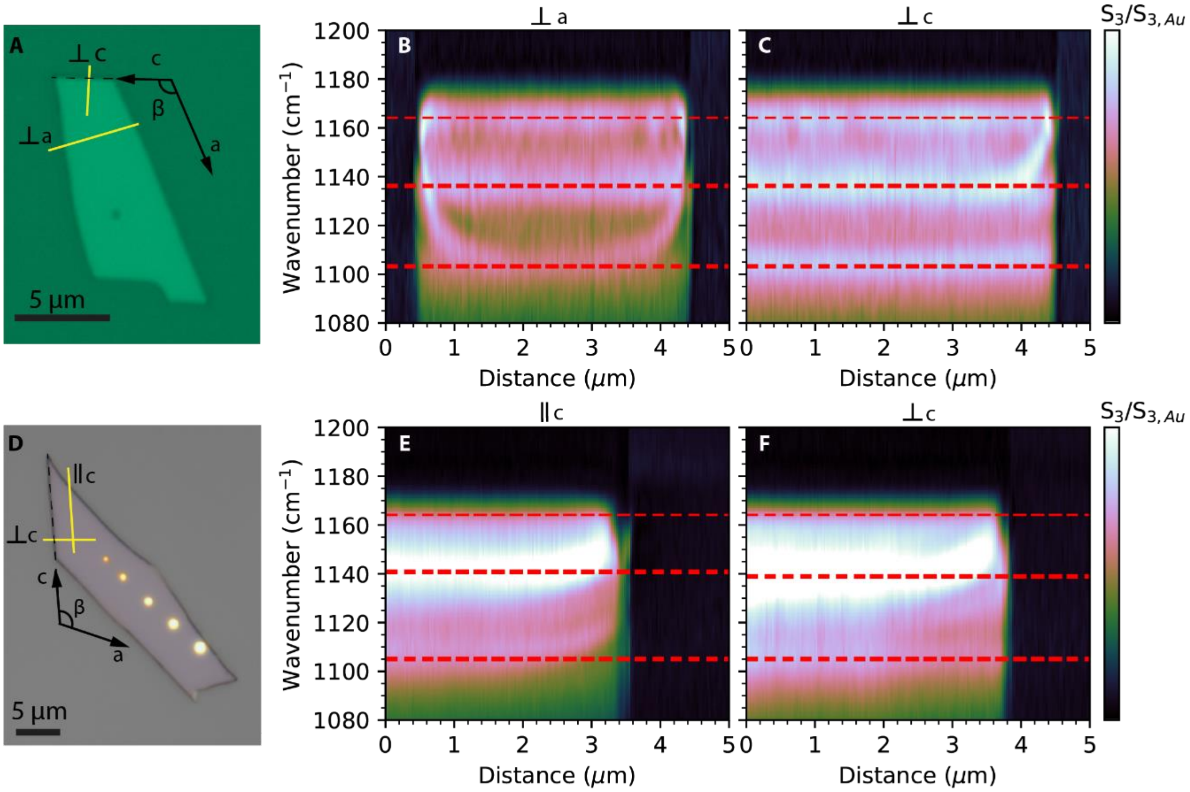

Fig. S10: nano-FTIR spectroscopic line scans of gypsum flakes in the range 1080-1200 cm^{-1}. (**A,D**) Optical images of gypsum flakes with a thickness of 75 and 150 nm, respectively. The yellow lines mark the corresponding nano-FTIR spectral line scans. (**B,C**) nano-FTIR line scans perpendicular to the *a* and *c* crystal axes respectively of the flake in (A). (**E,F**) nano-FTIR line scan parallel and perpendicular to the *c* crystal axis respectively of the flake in **(**D). In the thinnest flake we clearly identify the two RBs mentioned in the main text, reproduced here for comparison. In the thicker flake we can also identify the same regions. Moreover, the TO phonon at 1135 cm^{-1} is much stronger in relative terms than in the thinner flake and slightly shifted to higher frequencies. In the line scan parallel to the *c* axis, we observe fringes in both RBs, whereas if the scan is performed perpendicular to it, the fringe is observed only in RB_2, in agreement with the measurements in the thinner flake.

REFERENCES AND NOTES

1. S. Dai, Z. Fei, Q. Ma, A. S. Rodin, M. Wagner, A. S. McLeod, M. K. Liu, W. Gannett, W. Regan, K. Watanabe, T. Taniguchi, M. Thiemens, G. Dominguez, A. H. C. Neto, A. Zettl, F. Keilmann, P. Jarillo-Herrero, M. M. Fogler, D. N. Basov, Tunable phonon polaritons in atomically thin van der Waals crystals of boron nitride. *Science* **343**, 1125–1129 (2014).

2. P. Li, M. Lewin, A. V. Kretinin, J. D. Caldwell, K. S. Novoselov, T. Taniguchi, K. Watanabe, F. Gaussmann, T. Taubner, Hyperbolic phonon-polaritons in boron nitride for near-field optical imaging and focusing. *Nat. Commun.* **6**, 7507 (2015).

3. A. J. Giles, S. Dai, I. Vurgaftman, T. Hoffman, S. Liu, L. Lindsay, C. T. Ellis, N. Assefa, I. Chatzakis, T. L. Reinecke, J. G. Tischler, M. M. Fogler, J. H. Edgar, D. N. Basov, J. D. Caldwell, Ultralow-loss polaritons in isotopically pure boron nitride. *Nat. Mater.* **17**, 134–139 (2018).

4. I.-H. Lee, M. He, X. Zhang, Y. Luo, S. Liu, J. H. Edgar, K. Wang, P. Avouris, T. Low, J. D. Caldwell, S.-H. Oh, Image polaritons in boron nitride for extreme polariton confinement with low losses. *Nat. Commun.* **11**, 3649 (2020).

5. S. G. Menabde, S. Boroviks, J. Ahn, J. T. Heiden, K. Watanabe, T. Taniguchi, T. Low, D. K. Hwang, N Asger Mortensen, M. S. Jang, Near-field probing of image phonon-polaritons in hexagonal boron nitride on gold crystals. *Sci. Adv.* **8**, eabn0627 (2022).

6. J. D. Caldwell, A. V. Kretinin, Y. Chen, V. Giannini, M. M. Fogler, Y. Francescato, C. T. Ellis, J. G. Tischler, C. R. Woods, A. J. Giles, M. Hong, K. Watanabe, T. Taniguchi, S. A. Maier, K. S. Novoselov, Sub-diffractional volume-confined polaritons in the natural hyperbolic material hexagonal boron nitride. *Nat. Commun.* **5**, 5221 (2014).

7. W. Ma, P. Alonso-González, S. Li, A. Y. Nikitin, J. Yuan, J. Martín-Sánchez, J. Taboada-Gutiérrez, I. Amenabar, P. Li, S. Vélez, C. Tollan, Z. Dai, Y. Zhang, S. Sriram, K. Kalantar-Zadeh, S.-T. Lee, R. Hillenbrand, Q. Bao, In-plane anisotropic and ultra-low-loss polaritons in a natural van der Waals crystal. *Nature* **562**, 557–562 (2018).

8. Z. Zheng, N. Xu, S. L. Oscurato, M. Tamagnone, F. Sun, Y. Jiang, Y. Ke, J. Chen, W. Huang, W. L. Wilson, A. Ambrosio, S. Deng, H. Chen, A mid-infrared biaxial hyperbolic van der Waals crystal. *Sci. Adv.* **5**, eaav8690 (2019).

9. G. Álvarez-Pérez, T. G. Folland, I. Errea, J. Taboada-Gutiérrez, J. Duan, J. Martín-Sánchez, A. I. F. Tresguerres-Mata, J. R. Matson, A. Bylinkin, M. He, W. Ma, Q. Bao, J. I. Martín, J. D. Caldwell, A. Y. Nikitin, P. Alonso-González, Infrared permittivity of the biaxial van der Waals semiconductor α-MoO_3 from near- and far-field correlative studies. *Adv. Mater.* **32**, 1908176 (2020).

10. Y. Wu, Q. Ou, Y. Yin, Y. Li, W. Ma, W. Yu, G. Liu, X. Cui, X. Bao, J. Duan, G. Álvarez-Pérez, Z. Dai, B. Shabbir, N. Medhekar, X. Li, C.-M. Li, P. Alonso-González, Q. Bao, Chemical switching of low-loss phonon polaritons in α-MoO_3 by hydrogen intercalation. *Nat. Commun.* **11**, 2646 (2020).

11. P. Clauws, J. Vennik, Lattice vibrations of V2O5. Determination of TO and LO frequencies from infrared reflection and transmission. *Phys. Status Solidi B Basic Res.* **76**, 707–713 (1976).

12. S. Sucharitakul, G. Ye, W. R. L. Lambrecht, C. Bhandari, A. Gross, R. He, H. Poelman, X. P. A. Gao, V_2O_5: A 2D van der Waals oxide with strong in-plane electrical and optical anisotropy. *ACS Appl. Mater. Interfaces* **9**, 23949–23956 (2017).

13. J. Taboada-Gutiérrez, G. Álvarez-Pérez, J. Duan, W. Ma, K. Crowley, I. Prieto, A. Bylinkin, M. Autore, H. Volkova, K. Kimura, T. Kimura, M.-H. Berger, S. Li, Q. Bao, X. P. A. Gao, I. Errea, A. Y. Nikitin, R. Hillenbrand, J. Martín-Sánchez, P. Alonso-González, Broad spectral tuning of ultra-low-loss polaritons in a van der Waals crystal by intercalation. *Nat. Mater.* **19**, 964–968 (2020).

14. S. Adachi, *Optical Properties of Crystalline and Amorphous Semiconductors* (Springer, 1999); http://link.springer.com/10.1007/978-1-4615-5241-3.

15. G. Álvarez-Pérez, J. Duan, J. Taboada-Gutiérrez, Q. Ou, E. Nikulina, S. Liu, J. H. Edgar, Q. Bao, V. Giannini, R. Hillenbrand, J. Martín-Sánchez, A. Y. Nikitin, P. Alonso-González, Negative reflection of nanoscale-confined polaritons in a low-loss natural medium. *Sci. Adv.* **8**, eabp8486 (2022).

16. J. Duan, G. Álvarez-Pérez, A. I. F. Tresguerres-Mata, J. Taboada-Gutiérrez, K. V. Voronin, A. Bylinkin, B. Chang, S. Xiao, S. Liu, J. H. Edgar, J. I. Martín, V. S. Volkov, R. Hillenbrand, J. Martín-

Sánchez, A. Y. Nikitin, P. Alonso-González, Planar refraction and lensing of highly confined polaritons in anisotropic media. *Nat. Commun.* **12**, 4325 (2021).

17. H. Hu, N. Chen, H. Teng, R. Yu, M. Xue, K. Chen, Y. Xiao, Y. Qu, D. Hu, J. Chen, Z. Sun, P. Li, F. J. G. de Abajo, Q. Dai, Gate-tunable negative refraction of mid-infrared polaritons. *Science* **379**, 558–561 (2023).

18. P. Li, G. Hu, I. Dolado, M. Tymchenko, C.-W. Qiu, F. J. Alfaro-Mozaz, F. Casanova, L. E. Hueso, S. Liu, J. H. Edgar, S. Vélez, A. Alu, R. Hillenbrand, Collective near-field coupling and nonlocal phenomena in infrared-phononic metasurfaces for nano-light canalization. *Nat. Commun.* **11**, 3663 (2020).

19. J. Duan, G. Álvarez-Pérez, C. Lanza, K. Voronin, A. I. F. Tresguerres-Mata, N. Capote-Robayna, J. Álvarez-Cuervo, A. Tarazaga Martín-Luengo, J. Martín-Sánchez, V. S. Volkov, A. Y. Nikitin, P. Alonso-González, Multiple and spectrally robust photonic magic angles in reconfigurable α-MoO_3 trilayers. *Nat. Mater.* **22**, 867–872 (2023).

20. S. Dai, Q. Ma, T. Andersen, A. S. Mcleod, Z. Fei, M. K. Liu, M. Wagner, K. Watanabe, T. Taniguchi, M. Thiemens, F. Keilmann, P. Jarillo-Herrero, M. M. Fogler, D. N. Basov, Subdiffractional focusing and guiding of polaritonic rays in a natural hyperbolic material. *Nat. Commun.* **6**, 6963 (2015).

21. M. Autore, P. Li, I. Dolado, F. J. Alfaro-Mozaz, R. Esteban, A. Atxabal, F. Casanova, L. E. Hueso, P. Alonso-González, J. Aizpurua, A. Y. Nikitin, S. Vélez, R. Hillenbrand, Boron nitride nanoresonators for phonon-enhanced molecular vibrational spectroscopy at the strong coupling limit. *Light Sci. Appl.* **7**, 17172–17172 (2018).

22. A. Bylinkin, M. Schnell, M. Autore, F. Calavalle, P. Li, J. Taboada-Gutièrrez, S. Liu, J. H. Edgar, F. Casanova, L. E. Hueso, P. Alonso-Gonzalez, A. Y. Nikitin, R. Hillenbrand, Real-space observation of vibrational strong coupling between propagating phonon polaritons and organic molecules. *Nat. Photon.* **15**, 197–202 (2021).

23. C. Xu, H. Cai, D.-W. Wang, Vibrational strong coupling between Tamm phonon polaritons and organic molecules. *J. Opt. Soc. Am. B* **38**, 1505–1509 (2021).

24. I. Dolado, C. Maciel-Escudero, E. Nikulina, E. Modin, F. Calavalle, S. Chen, A. Bylinkin, F. J. Alfaro-Mozaz, J. Li, J. H. Edgar, F. Casanova, S. Vélez, L. E. Hueso, R. Esteban, J. Aizpurua, R. Hillenbrand, Remote near-field spectroscopy of vibrational strong coupling between organic molecules and phononic nanoresonators. *Nat. Commun.* **13**, 6850 (2022).

25. A. Bylinkin, F. Calavalle, M. Barra-Burillo, R. V. Kirtaev, E. Nikulina, E. Modin, E. Janzen, J. H. Edgar, F. Casanova, L. E. Hueso, V. S. Volkov, P. Vavassori, I. Aharonovich, P. Alonso-Gonzalez, R. Hillenbrand, A. Y. Nikitin, Dual-band coupling of phonon and surface plasmon polaritons with vibrational and electronic excitations in molecules. *Nano Lett.* **23**, 3985–3993 (2023).

26. N. C. Passler, X. Ni, G. Hu, J. R. Matson, G. Carini, M. Wolf, M. Schubert, A. Alù, J. D. Caldwell, T. G. Folland, A. Paarmann, Hyperbolic shear polaritons in low-symmetry crystals. *Nature* **602**, 595–600 (2022).

27. J. Matson, S. Wasserroth, X. Ni, M. Obst, K. Diaz-Granados, G. Carini, E. M. Renzi, E. Galiffi, T. G. Folland, L. M. Eng, J. Michael Klopf, S. Mastel, S. Armster, V. Gambin, M. Wolf, S. C. Kehr, A. Alù, A. Paarmann, J. D. Caldwell, Controlling the propagation asymmetry of hyperbolic shear polaritons in beta-gallium oxide. *Nat. Commun.* **14**, 5240 (2023).

28. G. Hu, W. Ma, D. Hu, J. Wu, C. Zheng, K. Liu, X. Zhang, X. Ni, J. Chen, X. Zhang, Q. Dai, J. D. Caldwell, A. Paarmann, A. Alù, P. Li, C.-W. Qiu, Real-space nanoimaging of hyperbolic shear polaritons in a monoclinic crystal. *Nat. Nanotechnol.* **18**, 64–70 (2023).

29. E. M. Renzi, E. Galiffi, X. Ni, A. Alù, Hyperbolic Shear Metasurfaces. *Phys. Rev. Lett.* **132**, 263803 (2024).

30. R. Claus, Polariton dispersion and crystal optics in monoclinic materials. *Phys. Status Solidi* **88**, 683–688 (1978),.

31. A. Krasnok, A. Alù, Low-Symmetry Nanophotonics. *ACS Photon.* **9**, 2–24 (2022).

32. E. Galiffi, G. Carini, X. Ni, G. Álvarez-Pérez, S. Yves, E. M. Renzi, R. Nolen, S. Wasserroth, M. Wolf, P. Alonso-Gonzalez, A. Paarmann, A. Alù, Extreme light confinement and control in low-symmetry phonon-polaritonic crystals. *Nat. Rev. Mater.* **9**, 9–28 (2024).

33. S. Yves, E. Galiffi, X. Ni, E. M. Renzi, A. Alù, Twist-induced hyperbolic shear metasurfaces. *Phys. Rev. X* **14**, 021031 (2024).

34. N. Lushnikova, L. Dvorkin, "25 - Sustainability of gypsum products as a construction material," in *Sustainability of Construction Materials (Second Edition)*, J. M. Khatib, Ed. (Woodhead Publishing, 2016), pp. 643–681.

35. V. K. Singh, "13 - Types of gypsum and set regulation of cement," in *The Science and Technology of Cement and Other Hydraulic Binders*, V. K. Singh, Ed. (Woodhead Publishing, 2023), pp. 467–497.

36. S. Palacio, J. Azorín, G. Montserrat-Martí, J. P. Ferrio, The crystallization water of gypsum rocks is a relevant water source for plants. *Nat. Commun.* **5**, 4660 (2014).

37. B. F. Pedersen, D. Semmingsen, Neutron diffraction refinement of the structure of gypsum, $CaSO_4.2H_2O$. *Acta Crystallogr. B* **38**, 1074–1077 (1982).

38. J. R. Aronson, A. G. Emslie, E. V. Miseo, E. M. Smith, P. F. Strong, Optical constants of monoclinic anisotropic crystals: gypsum. *Appl. Opt.* **22**, 4093–4098 (1983).

39. G. Anbalagan, S. Mukundakumari, K. S. Murugesan, S. Gunasekaran, Infrared, optical absorption, and EPR spectroscopic studies on natural gypsum. *Vib. Spectrosc.* **50**, 226–230 (2009).

40. W. Chen, W. Zhao, Y. Wu, Y. Wang, B. Zhang, F. Li, Q. Chen, Z. Qi, Z. Xu, Origin of gypsum growth habit difference as revealed by molecular conformations of surface-bound citrate and tartrate. *CrstEngComm* **20**, 3581–3589 (2018).

41. J. C. C. Santos, F. R. Negreiros, L. S. Pedroza, G. M. Dalpian, P. B. Miranda, Interaction of water with the gypsum (010) surface: Structure and dynamics from nonlinear vibrational spectroscopy and ab initio molecular dynamics. *J. Am. Chem. Soc.* **140**, 17141–17152 (2018).

42. N. Krishnamurthy, V. Soots, Raman spectrum of gypsum. *Can. J. Phys.* **49**, 885–896 (1971).

43. B. J. Berenblut, P. Dawson, G. R. Wilkinson, The Raman spectrum of gypsum. *Spectrochim. Acta A Mol. Biomol. Spectrosc.* **27**, 1849–1863 (1971).

44. E. Knittle, W. Phillips, Q. Williams, An infrared and Raman spectroscopic study of gypsum at high pressures. *Phys. Chem. Min.* **28**, 630–640 (2001).

45. H. Takahashi, I. Maehara, N. Kaneko, Infrared reflection spectra of gypsum. *Spectrochim. Acta A Mol. Biomol. Spectrosc.* **39**, 449–455 (1983).

46. N. Prieto-Taboada, O. Gómez-Laserna, I. Martínez-Arkarazo, M. Á. Olazabal, J. M. Madariaga, Raman spectra of the different phases in the $CaSO_4$–H_2O system. *Anal. Chem.* **86**, 10131–10137 (2014).

47. W. F. Cole, C. J. Lancucki, Hydrogen bonding in gypsum. *Nat. Phys. Sci.* **242**, 104–105 (1973).

48. T. G. Mayerhöfer, V. Ivanovski, J. Popp, Dispersion analysis of non-normal reflection spectra from monoclinic crystals. *Vib. Spectrosc.* **63**, 396–403 (2012).

49. E. E. Koch, A. Otto, K. L. Kliewwer, Reflection spectroscopy on monoclinic crystals. *Chem. Phys.* **3**, 362–369 (1974).

50. J. Chen, M. Badioli, P. Alonso-González, S. Thongrattanasiri, F. Huth, J. Osmond, M. Spasenović, A. Centeno, A. Pesquera, P. Godignon, A. Zurutuza Elorza, N. Camara, F. J. G. de Abajo, R. Hillenbrand, F. H. L. Koppens, Optical nano-imaging of gate-tunable graphene plasmons. *Nature* **487**, 77–81 (2012).

51. Z. Fei, A. S. Rodin, G. O. Andreev, W. Bao, A. S. McLeod, M. Wagner, L. M. Zhang, Z. Zhao, M. Thiemens, G. Dominguez, M. M. Fogler, A. H. C. Neto, C. N. Lau, F. Keilmann, D. N. Basov, Gate-tuning of graphene plasmons revealed by infrared nano-imaging. *Nature* **487**, 82–85 (2012).

52. S. Chen, P. L. Leng, A. Konečná, E. Modin, M. Gutierrez-Amigo, E. Vicentini, B. Martín-García, M. Barra-Burillo, I. Niehues, C. Maciel Escudero, X. Y. Xie, L. E. Hueso, E. Artacho, J. Aizpurua, I. Errea, M. G. Vergniory, A. Chuvilin, F. X. Xiu, R. Hillenbrand, Real-space observation of ultraconfined in-plane anisotropic acoustic terahertz plasmon polaritons. *Nat. Mater.* **22**, 860–866 (2023).

53. S. Chen, A. Bylinkin, Z. Wang, M. Schnell, G. Chandan, P. Li, A. Y. Nikitin, S. Law, R. Hillenbrand, Real-space nanoimaging of THz polaritons in the topological insulator Bi2Se3. *Nat. Commun.* **13**, 1374 (2022).

54. N. C. Passler, A. Paarmann, Generalized 4 × 4 matrix formalism for light propagation in anisotropic stratified media: Study of surface phonon polaritons in polar dielectric heterostructures. *J. Opt. Soc. Am. B* **34**, 2128–2139 (2017).

55. K. L. Tsakmakidis, A. D. Boardman, O. Hess, 'Trapped rainbow' storage of light in metamaterials. *Nature* **450**, 397–401 (2007).

56. K. L. Tsakmakidis, O. Hess, R. W. Boyd, X. Zhang, Ultraslow waves on the nanoscale. *Science* **358**, eaan5196 (2017).

57. M. Klein, R. Binder, M. R. Koehler, D. G. Mandrus, T. Taniguchi, K. Watanabe, J. R. Schaibley, Slow light in a 2D semiconductor plasmonic structure. *Nat. Commun.* **13**, 6216 (2022).

58. A. Kumar, Y. J. Tan, N. Navaratna, M. Gupta, P. Pitchappa, R. Singh, Slow light topological photonics with counter-propagating waves and its active control on a chip. *Nat. Commun.* **15**, 926 (2024).

59. S. Dai, W. Fang, N. Rivera, Y. Stehle, B.-Y. Jiang, J. Shen, R. Y. Tay, C. J. Ciccarino, Q. Ma, D. Rodan-Legrain, P. Jarillo-Herrero, E. H. T. Teo, M. M. Fogler, P. Narang, J. Kong, D. N. Basov, Phonon polaritons in monolayers of hexagonal boron nitride. *Adv. Mater.* **31**, e1806603 (2019).

60. J. Duan, Y. Li, Y. Zhou, Y. Cheng, J. Chen, Near-field optics on flatland: From noble metals to van der Waals materials. *Adv. Phys. X* **4**, 1593051 (2019).

61. G. A. Ermolaev, K. V. Voronin, A. N. Toksumakov, D. V. Grudinin, I. M. Fradkin, A. Mazitov, A. S. Slavich, M. K. Tatmyshevskiy, D. I. Yakubovsky, V. R. Solovey, R. V. Kirtaev, S. M. Novikov, E. S. Zhukova, I. Kruglov, A. A. Vyshnevyy, D. G. Baranov, D. A. Ghazaryan, A. V. Arsenin, L. Martin-Moreno, V. S. Volkov, K. S. Novoselov, Wandering principal optical axes in van der Waals triclinic materials. *Nat. Commun.* **15**, 1552 (2024).

62. Z. Zheng, F. Sun, W. Huang, J. Jiang, R. Zhan, Y. Ke, H. Chen, S. Deng, Phonon polaritons in twisted double-layers of hyperbolic van der Waals Crystals. *Nano Lett.* **20**, 5301–5308 (2020).

63. G. Hu, Q. Ou, G. Si, Y. Wu, J. Wu, Z. Dai, A. Krasnok, Y. Mazor, Q. Zhang, Q. Bao, C.-W. Qiu, A. Alù, Topological polaritons and photonic magic angles in twisted α-MoO_3 bilayers. *Nature* **582**, 209–213 (2020).

64. C.-L. Zhou, X.-H. Wu, Y. Zhang, H.-L. Yi, M. Antezza, Polariton topological transition effects on radiative heat transfer. *Phys. Rev. B* **103**, 155404 (2021).

65. S. Li, J. Zhou, W. Du, Configurable topological phonon polaritons in twisted hBN metasurfaces. *Appl. Opt.* **60**, 5735–5741 (2021).

66. J. Duan, G. Álvarez-Pérez, K. V. Voronin, I. Prieto, J. Taboada-Gutiérrez, V. S. Volkov, J. Martín-Sánchez, A. Y. Nikitin, P. Alonso-González, Enabling propagation of anisotropic polaritons along forbidden directions via a topological transition. *Sci. Adv.* **7**, eabf2690 (2021).

67. T. Nörenberg, G. Álvarez-Pérez, M. Obst, L. Wehmeier, F. Hempel, J. M. Klopf, A. Y. Nikitin, S. C. Kehr, L. M. Eng, P. Alonso-González, T. V. A. G. de Oliveira, Germanium monosulfide as a natural platform for highly anisotropic THz polaritons. *ACS Nano* **16**, 20174–20185 (2022).

68. M. Lifshitz, Anomalies of electron characteristics of a metal in the high pressure region. *JETP* **11**, 1130 (1960).

69. G. Hu, A. Krasnok, Y. Mazor, C.-W. Qiu, A. Alù, Moiré hyperbolic metasurfaces. *Nano Lett.* **20**, 3217–3224 (2020).

70. Y. Zeng, Q. Ou, L. Liu, C. Zheng, Z. Wang, Y. Gong, X. Liang, Y. Zhang, G. Hu, Z. Yang, C.-W. Qiu, Q. Bao, H. Chen, Z. Dai, Tailoring topological transitions of anisotropic polaritons by interface engineering in biaxial crystals. *Nano Lett.* **22**, 4260–4268 (2022).

71. H. N. S. Krishnamoorthy, Z. Jacob, E. Narimanov, I. Kretzschmar, V. M. Menon, Topological transitions in metamaterials. *Science* **336**, 205–209 (2012).

72. J. S. Gomez-Diaz, M. Tymchenko, A. Alù, Hyperbolic plasmons and topological transitions over uniaxial metasurfaces. *Phys. Rev. Lett.* **114**, 233901 (2015).

73. L. L. Long, M. R. Querry, R. J. Bell, R. W. Alexander, Optical properties of calcite and gypsum in crystalline and powdered form in the infrared and far-infrared. *Infrared Phys.* **34**, 191–201 (1993).

74. Z. Wang, A. Bhattacharya, M. Yagmurcukardes, V. Kravets, P. Díaz-Núñez, C. Mullan, I. Timokhin, T. Taniguchi, K. Watanabe, A. N. Grigorenko, F. Peeters, K. S. Novoselov, Q. Yang, A. Mishchenko, Quantifying hydrogen bonding using electrically tunable nanoconfined water. *Nat. Commun.* **16**, 3447 (2025).

75. H. Friedrich, D. Wintgen, Physical realization of bound states in the continuum. *Phys. Rev. A* **31**, 3964–3966 (1985).

76. C. W. Hsu, B. Zhen, A. D. Stone, J. D. Joannopoulos, M. Soljačić, Bound states in the continuum. *Nat. Rev. Mater.* **1**, 16048 (2016).

77. H. Gupta, G. Venturi, T. Contino, E. Janzen, J. H. Edgar, F. De Angelis, A. Toma, A. Ambrosio, M. Tamagnone, Bound States in the Continuum and Long-Range Coupling of Polaritons in Hexagonal Boron Nitride Nanoresonators. *ACS Photon.* **11**, 4017–4026 (2024).

78. L. Nan, A. Mancini, T. Weber, G. L. Seah, E. Cortés, A. Tittl, S. A. Maier, Highly confined incident-angle-robust surface phonon polariton bound states in the continuum metasurfaces. arXiv:2403.18743 [physics.optics] (2024); https://doi.org/10.48550/arXiv.2403.18743.

79. J. Yang, K. Wang, L. Zhang, C. Zhang, A. Fan, Z. He, Z. Li, X. Han, F. Ling, P. Lu, Manipulating terahertz phonon-polariton in the ultrastrong coupling regime with bound states in the continuum. arXiv:2411.01914 [physics.optics] (2024). https://doi.org/10.48550/arXiv.2411.01914.

80. X. Wu, S. Zhang, J. Song, X. Deng, W. Du, X. Zeng, Y. Zhang, Z. Zhang, Y. Chen, Y. Wang, C. Jiang, Y. Zhong, B. Wu, Z. Zhu, Y. Liang, Q. Zhang, Q. Xiong, X. Liu, Exciton polariton condensation from bound states in the continuum at room temperature. *Nat. Commun.* **15**, 3345 (2024).

81. Y. Zhu, J. Hou, Q. Geng, B. Xue, Y. Chen, X. Chen, L. Ge, W. Wan, Storing light near an exceptional point. *Nat. Commun.* **15**, 8101 (2024).

82. Ş. K. Özdemir, S. Rotter, F. Nori, L. Yang, Parity–time symmetry and exceptional points in photonics. *Nat. Mater.* **18**, 783–798 (2019).

83. M.-A. Miri, A. Alù, Exceptional points in optics and photonics. *Science* **363**, eaar7709 (2019).

84. A. Li, H. Wei, M. Cotrufo, W. Chen, S. Mann, X. Ni, B. Xu, J. Chen, J. Wang, S. Fan, C.-W. Qiu, A. Alù, L. Chen, Exceptional points and non-Hermitian photonics at the nanoscale. *Nat. Nanotechnol.* **18**, 706–720 (2023).

85. H. Alaeian, J. A. Dionne, Parity-time-symmetric plasmonic metamaterials. *Phys. Rev. A* **89**, 033829 (2014).

86. R. Su, E. Estrecho, D. Biegańska, Y. Huang, M. Wurdack, M. Pieczarka, A. G. Truscott, T. C. H. Liew, E. A. Ostrovskaya, Q. Xiong, Direct measurement of a non-Hermitian topological invariant in a hybrid light-matter system. *Sci. Adv.* **7**, eabj8905 (2021).

87. Z. Guo, J. Jiang, Y. Wang, J. Alvarez-Cuervo, A. T. Martin-Luengo, S. Hu, J. Jiang, P. A. Gonzalez, J. Duan, H. Chen, Exceptional point empowered near-field routing of hyperbolic polaritons. *Sci. Bull.* **69**, 3491–3495 (2024).

88. Y. Xu, L. Li, H. Jeong, S. Kim, I. Kim, J. Rho, Y. Liu, Subwavelength control of light transport at the exceptional point by non-Hermitian metagratings. *Sci. Adv.* **9**, eadf3510 (2023).

89. J. Wiersig, Sensors operating at exceptional points: General theory. *Phys. Rev. A* **93**, 033809 (2016).

90. S. H. Park, S. Xia, S.-H. Oh, P. Avouris, T. Low, Accessing the exceptional points in a graphene plasmon–vibrational mode coupled system. *ACS Photon.* **8**, 3241–3248 (2021).

91. J.-H. Park, A. Ndao, W. Cai, L. Hsu, A. Kodigala, T. Lepetit, Y.-H. Lo, B. Kanté, Symmetry-breaking-induced plasmonic exceptional points and nanoscale sensing. *Nat. Phys.* **16**, 462–468 (2020).

92. G. Álvarez-Pérez, K. V. Voronin, V. S. Volkov, P. Alonso-González, A. Y. Nikitin, Analytical approximations for the dispersion of electromagnetic modes in slabs of biaxial crystals. *Phys. Rev. B* **100**, 235408 (2019).
